# Catalyst Identity Matters: Molecular Ag(I)–BIAN Complexes Outperform Ag Nanoparticles in High-Current CO_2_ Electroreduction

**DOI:** 10.1021/acsomega.6c02628

**Published:** 2026-09-02

**Authors:** Sankitkumar Vala, Dominik Krisch, David Pilz, Simon Offenthaler, Wolfgang Schöfberger

**Affiliations:** Institute of Organic Chemistry, Laboratory for Sustainable Chemistry and Catalysis (LSusCat), Johannes Kepler University (JKU), Altenberger Straße 69, Linz 4040, Austria

## Abstract

Redox-active ligands offer powerful opportunities to modulate catalytic reactivity while minimizing precious metal loading. Herein, we report the mechanochemical synthesis and comprehensive characterization of homoleptic silver­(I) bis­(arylimino)­acenaphthene (Ag–BIAN) complexes and their application in homogeneous and heterogeneous zero-gap CO_2_ electroreduction. The molecular catalysts exhibit pronounced ligand-centered redox activity, with two reversible reductions assigned to sequential BIAN-based electron uptake. Under a CO_2_ atmosphere, significant catalytic current enhancement is observed between the first and second ligand-centered reductions, consistent with activation of electrogenerated reduced species. In a zero-gap electrolyzer configuration, the Ag–BIAN complexes achieve Faradaic efficiencies for CO formation of 70–90% at current densities up to 300 mA cm^–2^. Notably, the tailored molecular design enables a tenfold reduction in silver loading compared to Ag nanoparticle benchmarks while maintaining high catalytic performance. The material cost of the catalysts amounts to only €7.5–9.8 g^–1^, substantially improving economic feasibility. These findings demonstrate that redox-active ligand frameworks can decouple catalytic activity from metal mass loading and provide a viable molecular alternative to heterogeneous silver systems for efficient CO_2_-to-CO conversion.

## Graphical abstract

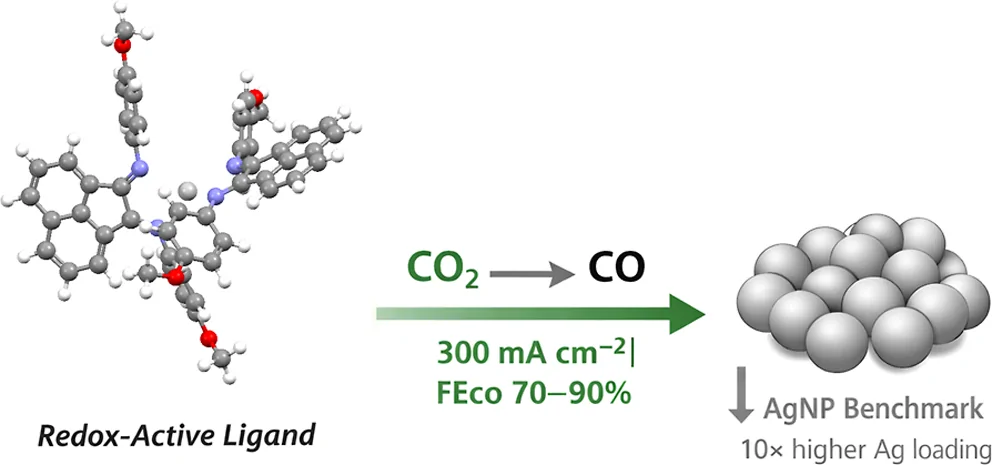

## Introduction

The growing environmental challenges of recent decades have intensified the need for sustainable energy technologies and effective strategies to mitigate rising atmospheric CO_2_ concentrations. The activation of abundant small molecules such as H_2_O,^1,2^ O_2_,^2–4^ N_2_,^5–7^ and CO_2_ ^8–12^ under mild, energy-efficient conditions represents a major focus of current catalysis research.

A major challenge in the activation of these small molecules is overcoming the substantial kinetic barriers that impede their transformation under mild conditions.^8^ Consequently, considerable research efforts have been devoted to the development of novel ligand frameworks whose metal complexes can facilitate the direct electrocatalytic reduction of CO_2_ into value-added products, including carbon monoxide, formate, methanol, ethanol, methane, ethylene, and acetate.^13–20^

While numerous nitrogen-donor ligand systems, including porphyrinoids, bipyridines, salen ligands, and pincer ligands, have been extensively investigated for CO_2_ electroreduction, the application of BIAN (*N*,*N*′-bis­(arylimino)­acenaphthene)-supported metal complexes in this field remains largely unexplored.^16^ To date, only a single family of BIAN-based electrocatalysts, namely, Re­(I)­(Ar-BIAN)­(CO)_3_Hal complexes (Ar = phenyl, mesityl, or 2,6-diisopropyl; Hal = Cl or Br), has been reported for the electrochemical reduction of CO_2_.^21,22^ Under homogeneous conditions in acetonitrile containing 2% H_2_O, the most active member of this series, Re­(I)­(2,6-diisopropyl-BIAN)­(CO)_3_Cl, produced CO with a maximum Faradaic efficiency of 24% after 4000 s of electrolysis at −1.85 V versus NHE.^21^

Silver–BIAN complexes are even less explored, with the limited number of available studies focusing primarily on their synthesis and structural characterization.^23–25^ Notably, none of the reported silver–BIAN compounds have been investigated as catalysts. In the present work, we address this gap by examining molecular silver­(I) bis-BIAN complexes bearing electronically tunable aryl substituents as electrocatalysts for the reduction of CO_2_. This study expands the scope of BIAN-supported catalysts and provides new insights into the catalytic potential of silver–BIAN systems for carbon dioxide conversion.

In the present study, molecular silver­(I) bis-BIAN complexes featuring electron-donating substituents were investigated as catalysts for electrochemical CO_2_ reduction. The redox-active, homoleptic silver­(I) bis-BIAN complexes were prepared mechanochemically and thoroughly characterized by spectroscopic and electrochemical methods. The electrocatalytic performance of the complexes was evaluated under homogeneous conditions in a single-compartment electrochemical cell and under heterogeneous conditions in a zero-gap electrochemical cell to assess their activity, selectivity, and stability toward CO_2_ reduction.

## Materials and Methods

### Materials

Chemicals and solvents were purchased from Alfa Aesar, BLDpharm, The Fuel Cell Store, Merck, Sigma-Aldrich, Strem Chemicals, Thermo Fisher Scientific, and VWR International and were used without further purification unless otherwise specified. Anhydrous solvents (THF and CH_3_CN) were obtained from a molecular sieve under a N_2_ atmosphere (MB-SPS-7, M. Braun Inertgas-Systeme). High-purity water (18 MΩ cm) was received from a Milli-Q Reference A+ purification system with a MillipakQ-40 filter unit (0.22 μm pore size, Merck). High-purity gases (argon 5.0, carbon dioxide 4.5 and helium 5.0) were sourced from Linde Gas.

### Methods

^1^H NMR and ^13^C NMR spectra were recorded on a Bruker AVIII 300 or a Bruker AVIII 500 MHz spectrometer in deuterated solvents: CDCl_3_, DMSO-*d*_6_ (both Sigma-Aldrich), and D_2_O (Eurisotop). Chemical shifts (δ) are reported in parts per million (ppm) referenced to the residual solvent signals for ^1^H and ^13^C unless otherwise noted.

UV/vis absorption spectra were acquired on a Varian Cary 300 Bio UV/vis spectrophotometer. The measurements were performed in CH_2_Cl_2_ and CH_3_CN by using standard 10 mm quartz cuvettes. FT-IR absorption spectra were obtained on a Bruker ALPHA II compact FT-IR spectrometer.

XPS spectra were measured on a Thermo Fisher Scientific Nexsa G2 Surface Analysis System, equipped with a monochromatic Al Kα source (*h*ν = 1486.6 eV). The pressure within the analysis chamber was maintained at ultrahigh vacuum (∼10^–9^ mbar). Powder samples were tightly pressed onto indium foil to ensure good electrical contact with the sample holder. XPS survey spectra were recorded over the binding energy range 0–1400 eV with 200 eV pass energy and 30 s dwell time. High-resolution XPS spectra of C 1s, N 1s, O 1s, S 2s, S 2p, S 3s, Ag 3d, and AgMNN for chemical-state analysis were recorded with 20 eV pass energy and 30 s dwell time. The binding energies were referenced to the C 1s signal at 285 eV to correct for possible charging effects. Quantitative elemental compositions (at %) were determined from the recorded survey spectra by peak area integration using instrument-specific atomic sensitivity factors. Data analysis, including peak fitting and background subtraction, was performed in Thermo Scientific Avantage Software. HRMS-ESI spectra were recorded on an Agilent 6520 Q-TOF mass spectrometer with an ESI source, Agilent G1607A coaxial sprayer, and Thermo Fisher Scientific LTQ Orbitrap XL with an Ion Max API source in positive mode. Samples were prepared as particle-free solutions in CH_2_Cl_2_ or CH_3_CN.

Product analysis, conducted via Gas-Chromatograph Nexis GC-2030 by Shimadzu. During the electrolysis in the closed cell, 250 μL of the headspace gas was taken for the quantification of evolved gas. CO and H_2_ were detected during the electrolysis in the cathodic region. For quantification purposes, the peak area was converted into a gas volume using the calibration curve.

The Faradaic efficiency (FE %) of the gas products can be quantified following this formula,

$$FE_{i}=\frac{z_{i}\cdot n_{i}\cdot F}{Q_{total}}=\frac{z_{i}\cdot \frac{f_{{CO}_{2}}\cdot t}{V_{loop}}\cdot x_{i}}{Q_{total}}\cdot F$$

where *z*_i_, *n*_i_, *Q*_total_, *f*_CO2_, *t*, *V*_loop_, *x*_i_, and *F* are the number of electrons involved in the reaction, the mole amount of the total product (mol), the total consumed charge (C), the flow rate of CO_2_ (5 mL min^–1^), the test time (60 min), the volume of the quantitative loop in GC instrument used for detection (0.25 mL), the mole amount of the product directly measured by GC instrument (mol), and the Faradaic constant (96485.33C mol^–1^), respectively.

### Synthesis and Characterization

In contrast to previously reported solvothermal routes to Ag–BIAN complexes,^26–34^ we have developed a two-step mechanochemical synthesis protocol to obtain **Ag1**–**Ag4**. Acenaphthenequinone was combined with a substoichiometric amount of acetic acid (25 mol %) at room temperature, followed by the addition of a slight excess of either 4-(methylthio)­aniline, 4-(*n*-hexylthio)­aniline, *p*-anisidine, or 4-(*n*-hexyloxy)­aniline. Milling was performed at 30 Hz in a 10 mL stainless steel grinding jar containing ten 2 mm stainless steel balls at room temperature for 30 min, affording the free BIAN ligands. The crude products were extracted with DCM and 0.1 M aqueous HCl. Following this, the organic phase was washed with deionized water, dried over Na_2_SO_4_, and concentrated under reduced pressure. The isolated yields of the ligands (**L1**–**L4**) were 89–94%.^32^ The homoleptic silver­(I) bis-BIAN complexes were prepared mechanochemically by treatment of AgBF_4_ with 2 equiv of the respective BIAN ligand. The bis-chelated complexes [Ag­(BIAN)_2_]­BF_4_ (**Ag1**–**Ag4**) were isolated as orange–red powders in excellent yields (up to 97%; Scheme 1).

**Scheme 1:**
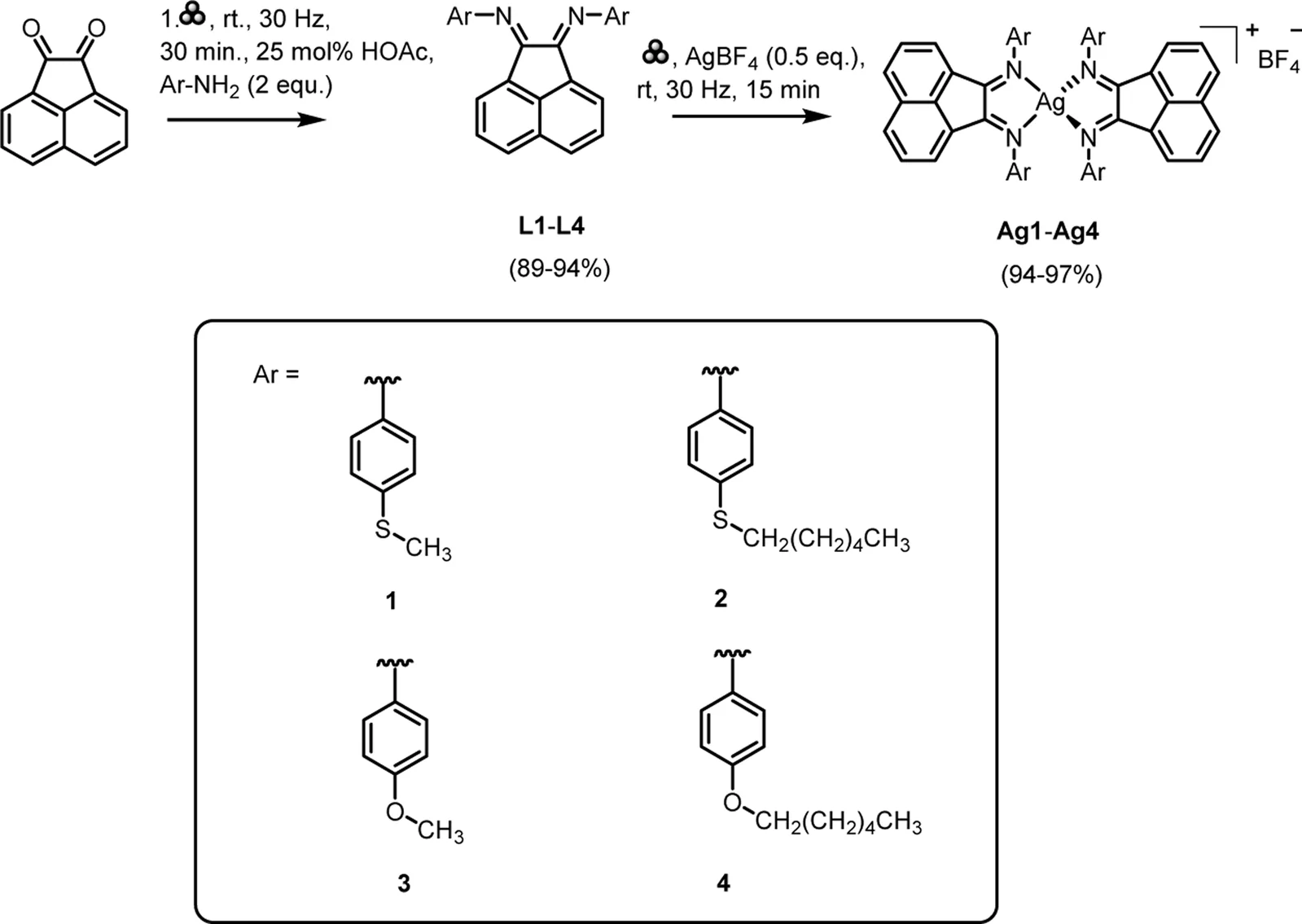
Mechanochemical Synthesis and Chemical Structures of the Ag­(I) Bis-BIAN Complexes **Ag1**–**Ag4**

Novel BIAN ligands and all silver complexes have been fully characterized by ^1^H and ^13^C NMR, ESI-HRMS, and UV–vis spectroscopy (Figures S1–S20). Due to the multifarious substitution patterns and consequential polarities of the products, however, multiple solvents had to be utilized for these measurements, limiting the direct comparability of some of the obtained data. Solution NMR experiments in CDCl_3_ and CD_3_CN confirmed that the investigated silver catalysts all exhibit diamagnetic properties, as expected for d^10^ metal complexes.

UV–vis spectroscopy revealed that despite metalation, no intense long-wavelength metal-to-ligand charge-transfer (MLCT) processes occur in the prepared complexes **Ag1**–**Ag4** and, predominantly, the intra-ligand-centered π–π* and *n*–π* transitions of the aryl, acenaphthene, and imine moieties are observed in acetonitrile solutions.^25,28,29^ Nevertheless, these absorption bands are altered upon coordination to a varying degree, indicating at least some participation of the Ag orbitals with the aforementioned excitations.^24^ The recorded ESI-HRMS spectra clearly display the expected monocationic complexes [Ag­(BIAN)_2_]^+^, corresponding to the loss of the BF_4_^–^ counterion during the ESI ionization process.

### Electrochemical Measurements

Cyclic voltammetry for electrochemical characterization and electrocatalytic CO_2_ reduction studies was performed at ambient temperature in acetonitrile using 0.1 M tetrabutylammonium hexafluorophosphate (TBAPF_6_) as the supporting electrolyte. Measurements were conducted in a single-compartment electrochemical cell (Pine Research Instrumentation) with 10 mL of solutions containing the 1 mM catalyst using a glassy carbon working electrode (geometric area = 0.28 cm^2^), a platinum counter electrode, and a non-aqueous Ag/AgCl quasi-reference electrode. Cyclic voltammograms were recorded at a scan rate of 100 mV s^–1^ over a potential range from −2.0 V to +1.5 V vs NHE, typically initiating the cathodic scans first. Before each measurement, the electrolyte was purged with Ar or CO_2_ for 15 min depending on the experiment. The electrocatalytic performance of complexes **Ag1–Ag4** and the corresponding ligands **L1–L4** was evaluated by cyclic voltammetry in Ar- and CO_2_-saturated acetonitrile, both in the absence and presence of 2% H_2_O as a proton source.

Potentials for the homogeneous electrochemical measurements are reported versus NHE, calculated from the ferrocene/ferrocenium half-wave potential determined in each electrolyte mixture as an internal standard.^35–37^

Zero-gap electrochemical measurements were performed using carbon cloth W1S1011 (area = 9 cm^2^) spray-coated with catalyst ink as the cathode, a high-porosity platinized titanium fiber felt (area = 9 cm^2^) spray-coated with iridium black ink as the anode, and a 40 μm PiperION membrane as the membrane separator (all Fuel Cell Store). The catalyst ink for the cathode included 2 mg mL^–1^ of **Ag2**, 2 mg mL^–1^ of ENSACO 250G carbon black, and 10 μL mL^–1^ of PiperION dispersion (5 wt %, Fuel Cell Store) in a 5:1 mixture of 2-propanol and 18 MΩ water. The catalyst ink for the anode included 2 mg mL^–1^ of iridium black and 10 μL mL^–1^ PiperION dispersion (5 wt %) in the same solvent mixture.

Inks were continuously ultrasonicated during spray-coating to maintain a homogeneous dispersion and loaded onto the respective substrates using a Worx MakerX Akku-Airbrush WX742.9 (0.10–0.12 MPa) on an 80 °C hot plate. Catalyst loadings of 0.5 mg cm^–2^ for **Ag2** and of 1.0 mg cm^–2^ for iridium black were determined gravimetrically by weighing the electrodes before and after spray-coating.

Measurements in a custom-built zero-gap electrochemical cell for **Ag2** were performed at 60 °C with a CO_2_ inlet stream of 100 mL min^–1^ at 50% RH and a 0.1 M CsOH anolyte flow rate of 100 mL min^–1^. Gaseous products were quantified by gas chromatography using a Shimadzu Nexis GC-2030 equipped with WCOT columns (Q-BOND and QS-BOND), barrier ionization discharge detectors (BIDs), and helium as the carrier gas. Both hydrogen and carbon monoxide were quantified by external standard calibration.

## Results and Discussion

Cyclic voltammograms (CVs) of the explored complexes under argon are primarily defined by the known distinctive redox non-innocence of BIANs.^22,24,29,38–45^ The first reduction generating a radical anion typically delocalized between the imine moieties is occurring at −1.0 V (Figure 1a,b). By further increasing the cathodic potential, a second reduction at the BIAN ligands to the pertinent diamines appears at potentials between −1.50 and −1.90 V.

**Figure 1:**
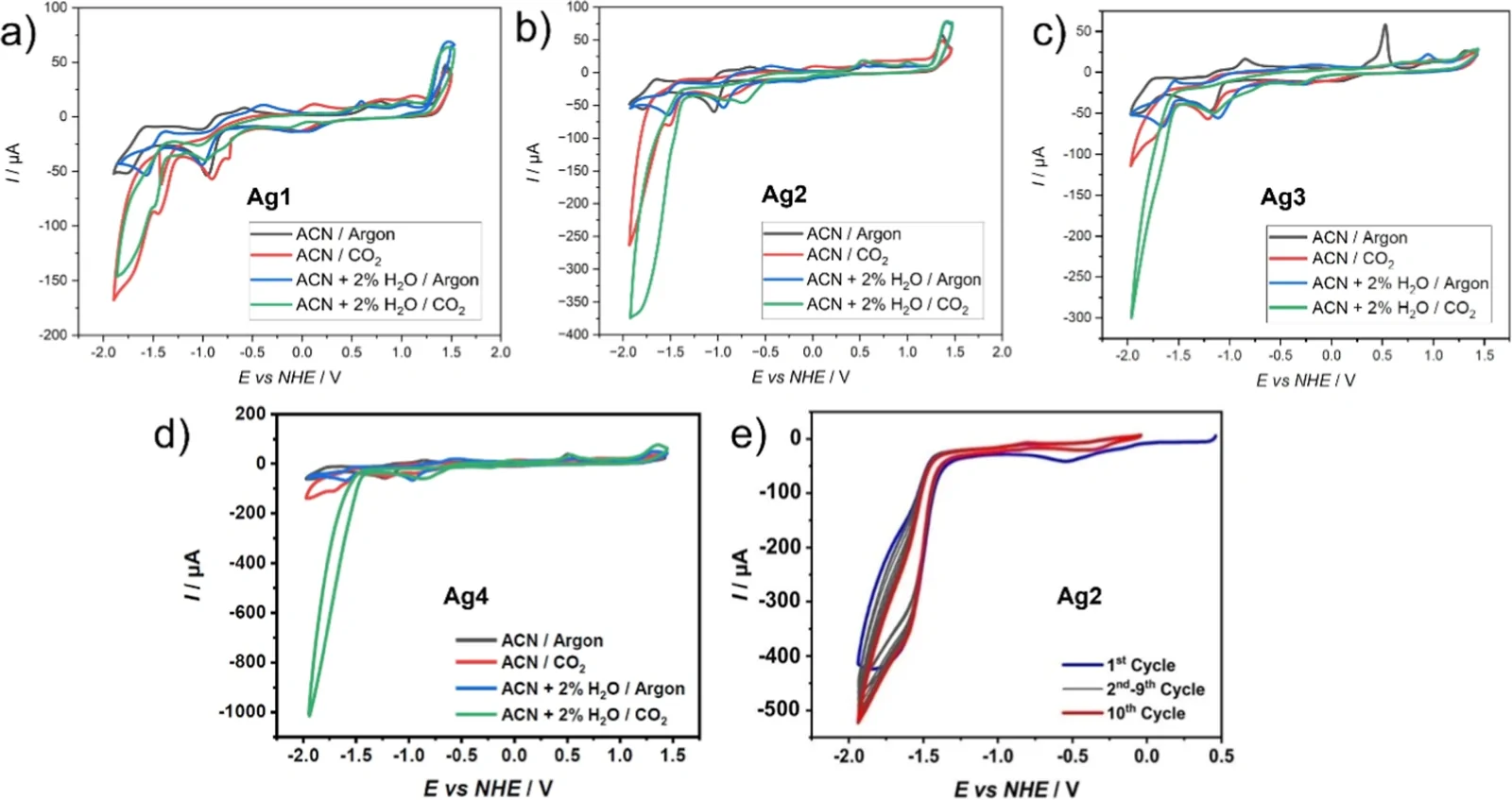
Homogeneous electrocatalysis of Ag­(I) bis-BIAN complexes **Ag1**–**Ag4**. Comparison of cyclic voltammograms of (a) **Ag1**, (b) **Ag2**, (c) **Ag3**, and (d) **Ag4** in ACN under argon (black), CO_2_ (red), in ACN with 2% H_2_O under argon (blue) and CO_2_ (green) at a scan rate of 100 mV s^–1^. (e) Successive cathodic scans of **Ag2** in ACN with 20% H_2_O under CO_2_ over ten cycles, indicating no decrease in catalytic current.

### Homogeneous Electrocatalysis

The electrocatalytic response of **Ag1–Ag4** toward CO_2_ reduction was subsequently evaluated by cyclic voltammetry under Ar and CO_2_ atmospheres. Upon introduction of CO_2_ into anhydrous acetonitrile solutions of the complexes, a pronounced increase in cathodic current is observed, emerging between the first and second ligand-centered reduction events. Concomitantly, these redox waves become irreversible, consistent with a follow-up chemical reaction of the electrogenerated reduced species with CO_2_. In the absence of an added proton source, **Ag1–Ag4** display a 3–5-fold enhancement in cathodic current at approximately −1.60 V under a CO_2_ atmosphere.

The addition of 2% H_2_O further increases the reductive currents for most complexes, whereas no increase is observed for **Ag1**. Among the four catalysts, **Ag2** exhibits the most significant current amplification, indicating a superior catalytic activity under the investigated conditions. Consequently, **Ag2** was selected for a subsequent in-depth study.

As depicted in Figure 1, the catalytic currents measured in organic solvents containing 2% water show a positive correlation with the increasing electron-donating character of the employed ligands. The difference between the currents observed under anhydrous conditions and those obtained in solvent–water mixtures can be attributed to the presence of proton sources, which enables alternative CO_2_ reduction pathways and consequently leads to distinct reaction kinetics.

In the absence of external proton donors, electrocatalytic CO_2_ reduction is generally proposed to proceed either through reductive disproportionation, yielding carbonate and CO, or, less commonly, through dimerization of the CO_2_ radical anion (CO_2_^•–^) to form oxalate. In contrast, the introduction of protons enables additional reaction pathways, resulting in a broader range of reduction products, including CO, formic acid, methanol, methane, and higher-value C_2+_ products such as ethylene, ethanol, and acetic acid.^13,14,16,46–49^

Importantly, none of the synthesized Ag­(I) bis-BIAN complexes exhibited significant catalytic H_2_ evolution in solvent mixtures containing 2% water under an argon atmosphere within the investigated potential window. This absence of competing proton reduction highlights their potential as selective CO reduction catalysts using water as an environmentally benign proton source.

Electrochemical investigations of the uncoordinated BIAN ligands **L1–L4** and the precursor complex AgBF_4_ further demonstrated that both the ligand framework and the coordinated silver center are required for the formation of catalytically active species. Neither the free ligands nor AgBF_4_ alone displayed CO_2_ reduction activity in ACN/H_2_O mixtures comparable to that of the corresponding chelated silver–BIAN complexes (Figures S21 and S22).

Although cyclic voltammetry of AgBF_4_ under CO_2_ in the presence of water revealed a pronounced increase in cathodic current at potentials below −1.5 V vs NHE when the full potential window was investigated, this current response was not maintained when the scan range was restricted to an anodic limit of +0.5 V vs NHE. This behavior contrasts with that of complexes **Ag1–Ag4**, which exhibit sustained catalytic currents during consecutive cathodic scans even in ACN containing 20% water (e.g., Figure 1c). Together with the results from heterogeneous CO_2_ reduction experiments performed with **Ag1–Ag4** (Figure 3), these observations indicate that the silver–BIAN complexes act as molecular electrocatalysts for CO_2_ reduction. In contrast, the response observed for AgBF_4_ is attributed to an undefined stoichiometric transformation of the precursor under reducing conditions, which is reversible upon oxidation at approximately +0.6 V vs NHE in ACN/20% H2O.

The cyclic voltammograms of **Ag1–Ag4** recorded in ACN/20% H_2_O mixtures show slightly decreased reversibility of the corresponding redox features. Nevertheless, electrochemical measurements performed under an argon atmosphere demonstrate that the investigated complexes do not promote significant hydrogen evolution from water electrolysis, as no substantial increase in cathodic current is observed under these conditions. The lower catalytic currents measured in CO_2_-saturated solvents compared to those obtained in the presence of 2% H_2_O are expected, considering the markedly lower solubility of CO_2_ in water relative to acetonitrile (0.033 M vs 0.28 M).^44^

To further explain the electronic changes occurring at **Ag2** and the free **L2** ligand upon cathodic polarization, a series of spectroscopic experiments were performed. In situ UV–vis spectroelectrochemistry (SEC/UV–vis) of **Ag2** revealed a progressive increase in absorbance at ∼265 nm upon stepwise cathodic polarization, accompanied by the emergence of a new absorption feature at ∼316 nm, consistent with the formation of the BIAN radical anion **[Ag2]** ^**•–**^ via ligand-centered reduction (Figure 2a,b). To independently validate the electrochemically generated species, chemical reduction of both **Ag2** and **L2** was performed using an excess of KC_8_ (potassium graphite intercalate, E° ≈−2.1 V) under a strict argon atmosphere. The UV–vis spectra of KC_8_-reduced **Ag2** and **L2** showed excellent agreement with those obtained by controlled-potential electrolysis at −1.4 V (Figure 2a,c), confirming that both chemical and electrochemical routes generate the same reduced species and that the observed spectral changes are intrinsic to ligand-centered reduction. The radical nature of the reduced species was unambiguously confirmed by EPR spectroscopy, where both KC_8_-reduced **Ag2** and **L2** exhibited signals in the organic radical region with g-values of g­(**[Ag2]** ^**•–**^) = 2.00319 and g­(**[L2** ^**•–**^ **]**) = 2.00309, respectively, calibrated against the BDPA reference standard (*g* = 2.0026) (Figure 2d). The proximity of these g-values to the free electron value (*g*_e_ = 2.00232) unequivocally confirms ligand-centered BIAN^•–^ radical anion character, in agreement with previous reports demonstrating that the iminopyridine-type nitrogen atoms of the BIAN backbone are primarily responsible for bearing the unpaired spin density upon one-electron reduction.^61^ The moderately narrower line width observed for **[Ag2]** ^**•**–^ (ΔB_pp_ = 2.54 G) relative to free **[L2** ^**•**–^] (ΔB_pp_ = 4.30 G) reflects the influence of the coordinated Ag­(I) center on the spin relaxation of the BIAN radical anion, while the retention of an organic radical g-value confirms that the Ag­(I) oxidation state remains intact upon reduction. Taken together, the convergent evidence from SEC/UV–vis, KC_8_ chemical reduction, and EPR spectroscopy establishes that electroreduction of **Ag2** proceeds exclusively through the redox non-innocent BIAN ligand framework, generating a persistent **[Ag2]** ^**•**–^ radical anion as the catalytically active species. The corresponding SEC/UV–vis and EPR data for the free **L2** ligand are provided in the Supporting Information (Figure S23), confirming that the same ligand-centered reduction mechanism operates in both the free ligand and the intact Ag­(I) complex.

**Figure 2:**
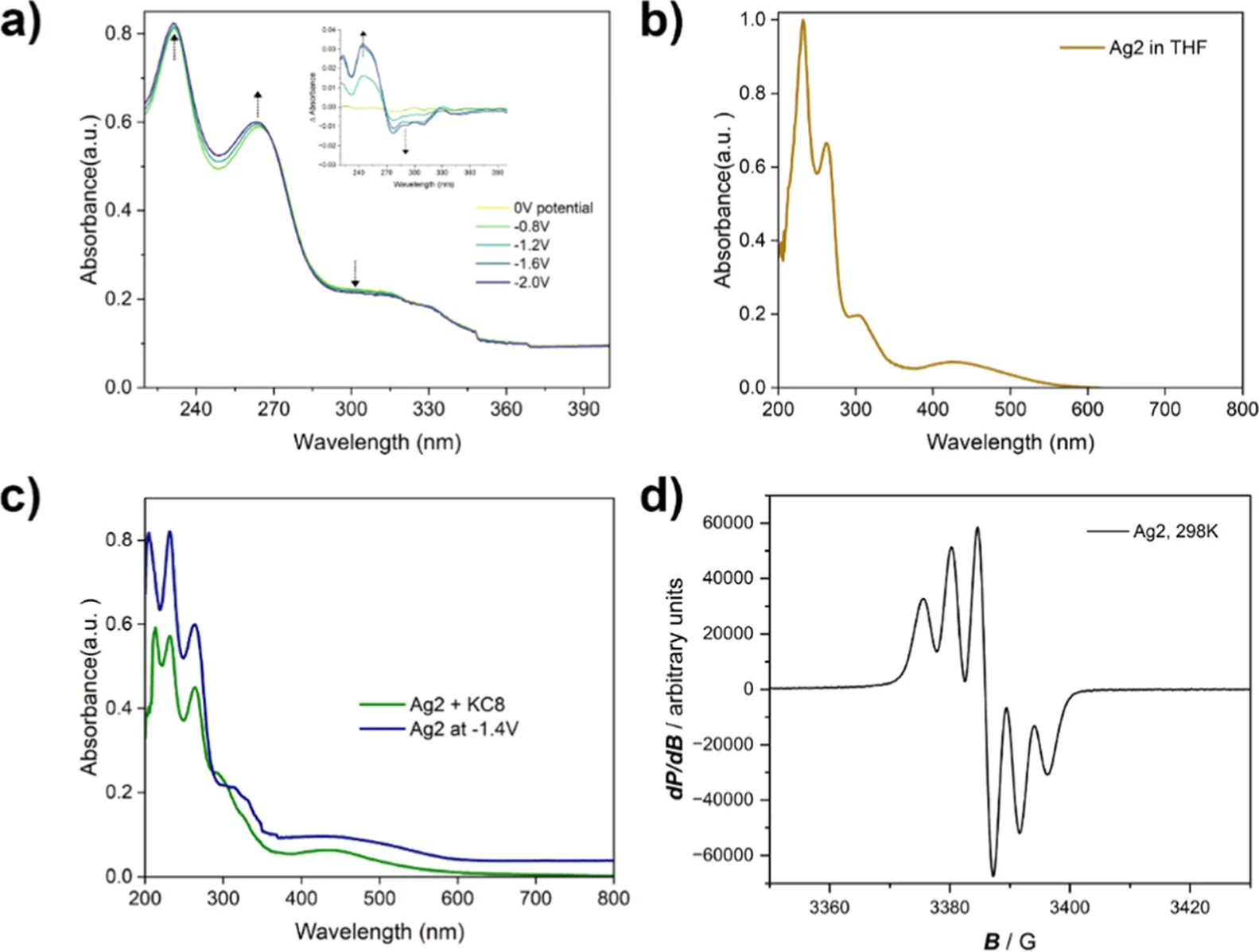
(a) In situ UV–vis spectroelectrochemistry of **Ag2** in THF at applied potentials from 0 V to −2.0 V, showing progressive increase in absorbance at ∼230 and ∼265 nm and decrease at ∼300 nm with increasing cathodic potential; the inset displays the differential spectra (ΔAbsorbance) highlighting the potential-dependent spectral evolution. (b) UV–vis absorption spectra of pristine **Ag2** in THF (gold). (c) Comparison of UV–vis spectra of **Ag2** reduced chemically with KC_8_ (green) and electrochemically at −1.4 V (blue) in THF, showing closely matching spectral profiles with characteristic absorption bands below 350 nm. (d) X-band EPR spectrum (first derivative, dP/dB) of chemically reduced **[Ag2]** ^**•**–^ generated by KC_8_ treatment (298 K, THF), recorded at *B* = 3340–3430 G (ν ≈9.51 GHz), showing a resolved hyperfine multiplet pattern centred at *g* ≈2.003.

As a final remark, due to the explored catalysts incorporating 18 electron metal centers, it seems unintuitive at first for their catalytic activity to stem from anything other than an outer sphere CO_2_ reduction mechanism.^16,49–51^ However, solid-state molecular structures, among others, of five coordinate bis-chelated Ag­(I) BIAN nitrate complexes, along with pentaligated [{MeB­[3-(Mes)­pz]_3_}­Ag­(C_2_H_4_)] (Mes = mesitylene; pz = pyrazolate), thus formally bearing 20 electrons at the metal center, have been reported.^24,52^ Hence, it appears coherent that CO_2_ would be able to ligate to the tetracoordinated silver center in the explored compounds **Ag1**–**Ag4**, generating a reactive intermediate during electrocatalytic reduction.

### Heterogeneous Electrocatalysis

To date, only a limited number of catalytic systems have successfully enabled a direct transition from homogeneous catalysis to industrially relevant gas diffusion electrode (GDE) architectures.^17,53^ In contrast to conventional H-type cells employing liquid electrolytes, GDE configurations minimize the diffusion distance of CO_2_ to the catalytic sites by facilitating gas-phase transport, thereby overcoming mass-transfer limitations and enabling current densities exceeding 50 mA cm^–2^ under aqueous operating conditions.^54^

Since **Ag2** has proven pristine catalytic activity and stability toward the generation of CO_2_R products, when employed homogeneously in high water content solvents, it was selected for preliminary studies on gas diffusion electrodes.

Chronopotentiometry potential–time curves showed stable potentials at 3.0, 3.45, and 4.1 V, confirming its stability. The Faradaic efficiencies range from 70 to 80% at 100–300 mA cm^–2^ under non-optimized conditions (Figure 3e,g) employing the above-mentioned procedures of catalyst ink and electrode preparation. Under optimized zero-gap conditions specifically tailored for **Ag2**, the catalyst maintains high selectivity for CO formation at industrially relevant current densities of 300 mA cm^–2^. As shown in Figure S24, the Faradaic efficiencies (FEs) for CO remain in the 70–90% range while achieving stable single-pass conversion (SPC) in a cell with a cathode area of 9 cm^2^. The improved performance compared to the initial configuration arises from deliberate optimization of the catalyst ink formulation, electrode preparation, and catalyst loading. This reduction in metal loading, combined with optimized catalyst-layer morphology and ionomer distribution, improves mass transport of CO_2_ and hydroxide ions within the gas-diffusion electrode. The refined microstructure enhances catalyst utilization and suppresses competing hydrogen evolution, thereby sustaining high CO selectivity even at elevated current densities. Overall, the optimized electrode architecture and ink engineering contribute synergistically to the improved electrocatalytic efficiency and stability observed for **Ag2** under high-rate CO_2_ electroreduction conditions.

**Figure 3:**
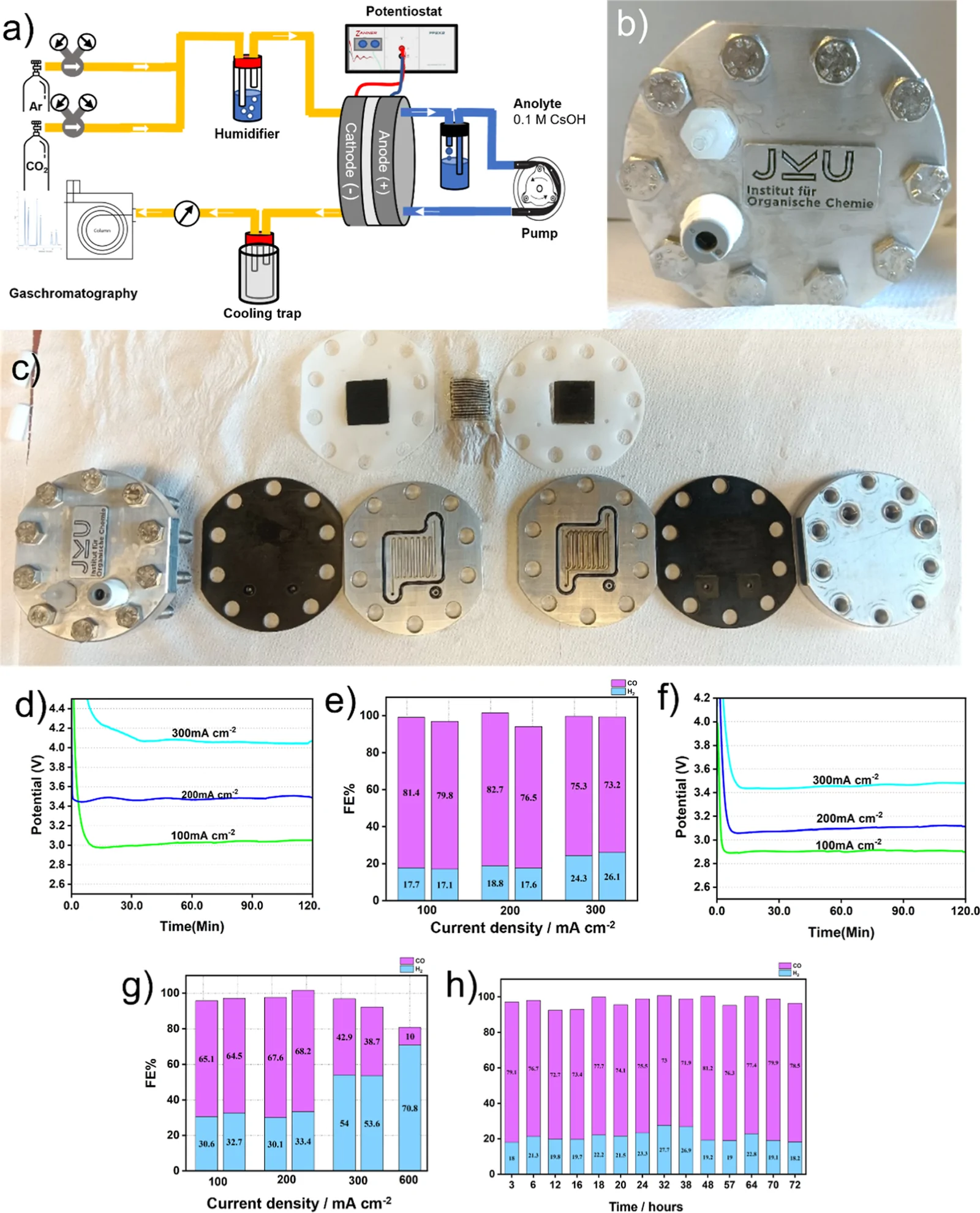
(a–c) CO_2_ electrolyzer setup operated at 60 °C with a CO_2_ inlet stream of 100 mL min^–1^ at 50% RH and a 0.1 M CsOH anolyte flow rate of 100 mL min^–1^. (d,e) Chronopotentiometric potential–time curves (E–t) and Faradaic efficiencies for H_2_ and CO of **Ag2** at current densities of 100, 200, and 300 mA cm^–2^. (f–g) For comparison, chronopotentiometric potential–time curves (E–t) and Faradaic efficiencies for H_2_ and CO of AgNPs. (h) Stability of **Ag2** at 300 mA cm^–2^ over 72 h, demonstrating selective and stable CO formation under prolonged electrolysis.

**Ag2** demonstrates superior performance compared to Ag nanoparticles (Figure 3f,g), which are commonly employed as a benchmark electrocatalyst for CO_2_-to-CO conversion, in terms of both catalyst loading and the overall electrocatalytic efficiency. This enhanced performance can be attributed to the non-innocent nature of the BIAN ligand within both the discrete molecular complexes. The ligand actively participates in the catalytic process through radical anion formation, facilitating improved electronic communication with the silver centers and promoting a more effective activation of the CO_2_ molecule. In the gas diffusion electrode environment, this ligand-assisted mechanism enables enhanced CO_2_ adsorption and activation, leading to higher catalytic activity and improved reaction kinetics relative to conventional Ag nanoparticle systems.

The obtained results highlight the promising potential of Ag–BIAN complexes as electrocatalysts for future CO_2_ reduction applications of industrial relevance. Previous studies have demonstrated that achieving optimal CO_2_ reduction performance requires the synergistic optimization of multiple factors, including catalyst design, reaction environment,^55,56^ electrode architecture,^57^ and operating parameters.^58–60^ Building upon these findings, future investigations will focus on the integration and optimization of BIAN-based catalysts on gas diffusion electrodes (GDEs) within state-of-the-art CO_2_ electrolyzer platforms.

## Conclusions

In summary, we have reported the mechanochemical synthesis and comprehensive characterization of four homoleptic silver­(I) bis-BIAN complexes and evaluated their performance as molecular electrocatalysts for CO_2_ reduction. The investigated complexes **Ag1–Ag4**, with the general formula [Ag­(I)­(BIAN)_2_]­BF_4_, can be readily synthesized through a two-step mechanochemical protocol from commercially available starting materials in excellent yields, without requiring rigorously dried solvents or extensive manipulations under Schlenk conditions.

All investigated complexes **Ag1–Ag4** demonstrated catalytic activity for the electrochemical reduction of CO_2_ upon application of cathodic potentials in ACN containing 2% H_2_O, establishing them as promising silver-based molecular CO_2_ reduction catalysts. Comparison of the catalytic current responses revealed that increasing the electron-donating character of the BIAN ligands enhances CO_2_ reduction performance. Furthermore, none of the investigated catalysts exhibited significant H_2_ evolution in aqueous electrolytes containing 2 vol % water under argon at potentials up to −2.0 V vs NHE, highlighting their selectivity toward CO_2_ reduction under these conditions.

The combined spectroscopic data therefore confirm that one-electron reduction of **Ag2** is ligand-centered, affording the **[Ag2]•** ^**–**^ BIAN radical anion as a persistent, spectroscopically characterized intermediate, and that the Ag­(I) center remains redox-innocent throughout the process. The identical spectral signatures obtained by both electrochemical and chemical (KC_8_) reduction routes confirm that the observed changes are intrinsic to the BIAN framework and not an artifact of the electrochemical conditions. The organic radical *g*-values (*g* = 2.003) and the characteristic UV–vis features developing upon cathodic polarization are fully consistent with delocalization of the unpaired electron over the BIAN π-system, in agreement with the established redox non-innocence of α-diimine ligands in late-transition-metal complexes. This ligand-centered electron storage capacity positions **[Ag2]** ^**•–**^ as a potent inner-sphere reductant primed for subsequent reactivity with CO_2_

In a zero-gap gas-diffusion electrolyzer operated at 60 °C, complex **Ag2** delivers high catalytic performance at current densities up to 300 mA cm^–2^, achieving Faradaic efficiencies for CO of 70–90%. Notably, CO selectivity is maintained with increasing current density, in contrast to typical silver nanoparticle benchmarks. Mechanistic investigations support a ligand-assisted pathway in which the reduced BIAN-ligand framework acts as an electron reservoir, enabling controlled two-electron transfer to coordinated CO_2_ while maintaining the Ag­(I) oxidation state. These findings demonstrate how molecularly engineered, redox-active ligand environments can outperform conventional metallic silver catalysts in gas-diffusion systems and provide a scalable platform for high-rate CO_2_ electroreduction.

These results greatly emphasize the potency of the reported Ag–BIAN complexes functionalized with electron-donating groups for electrochemical CO_2_ reduction, rendering them particularly appealing for further research and future applications. Notably, the tailored design of the novel Ag–BIAN complexes enables a tenfold lower silver loading compared to conventional AgNP-based systems. When benchmarked against the cost of commercially sourced silver nanoparticles (AgNPs), the material expenses associated with Ag–BIAN catalysts are significantly lower. Typical research-grade silver nanoparticle powders are listed at approximately €50–€200 per gram depending on particle size and purity. In contrast, **Ag1**–**Ag4** are €7.5-€9.8 per gram, representing at least an order of magnitude reduction in precious metal cost on a per-gram basis. Because the novel Ag–BIAN complexes also require approximately 10% of the silver loading relative to AgNPs to achieve comparable catalytic performance, the effective silver cost per unit of catalytic material is reduced by more than an order of magnitude overall. This dramatic cost advantage not only lowers the expenditure on silver metal itself but also improves the economic feasibility of scaling these catalysts for larger-scale applications, contributing to substantial total cost savings in both reagent procurement and catalyst preparation.

## Supplementary Material



## Notes

Materials and methods; synthesis procedures (4-*n*-hexylthioaniline, general procedure for the preparation of BIAN ligands, N^1^,N^2^-bis­(4-methylthiophenyl)­acenaphthylene-1,2-diimine (4-SMe-BIAN), N^1^,N^2^-bis­(4-hexylthiophenyl)­acenaphthylene-1,2-diimine (4-*n*-hexylthio-BIAN), N^1^,N^2^-bis­(4-methoxy-phenyl)­acenaphthylene-1,2-diimine­(4-OMe-BIAN), and N^1^,N^2^-bis­(4-*n*-hexyloxyphenyl)-acenaphthylene-1,2-diimine (4-*n*-hexyloxy-BIAN); synthesis of Ag­(I)–BIAN complexes, [Ag­(4-methylthio-BIAN)_2_]­BF_4_ (**Ag1**), [Ag­(4-*n*-hexylthio-BIAN)_2_]­BF_4_ (**Ag2**), [Ag­(4-OMe-BIAN)_2_]­BF_4_ (**Ag3**), and [Ag­(4-*n*-hexyloxy-BIAN)_2_]­BF_4_ (**Ag4**); Figure S1–S20: spectroscopic data; and Figure S21–S24: electrocatalysis data (PDF)
