# Supplementary material for: Catalyst Identity Matters: Molecular Ag(I)–BIAN Complexes Outperform Ag Nanoparticles in High-Current CO2 Electroreduction
Source: ACS Omega. 2026 Sep 2;11(36):53833–42. doi: 10.1021/acsomega.6c02628 (PMC13584162; doi:10.1021/acsomega.6c02628)
Supplement: Supplementary file 1 [file ao6c02628_si_001.pdf]

Catalyst Identity Matters: Molecular Ag(I)–BIAN Complexes Outperform Ag Nanoparticles in High-Current CO_2 Electroreduction

Sankitkumar Vala[a], Dominik Krisch[a], David Pilz[a], Simon Offenthaler[a], and Wolfgang Schöfberger*[a]

[a] Institute of Organic Chemistry, Laboratory for Sustainable Chemistry and Catalysis (LSusCat), Johannes Kepler University (JKU), Altenberger Straße 69, 4040 Linz, Austria.

Corresponding Authors: wolfgang.schoefberger@jku.at

Keywords: Ag(I)-bis(arylimino)acenaphthene • electrochemical CO_2 reduction • H-type electrochemical cell • zero-gap electrochemical cell.

Table of Contents

Materials and Methods 2

Synthesis procedures 2

4-*n*-hexylthioaniline 2

General Procedure for the Preparation of BIAN Ligands 3

N[1],*N*[2]-bis(4-methylthiophenyl)acenaphthylene-1,2-diimine (4-SMe-BIAN) 3

N[1],*N*[2]-bis(4-hexylthiophenyl)acenaphthylene-1,2-diimine (4-*n*-hexylthio-BIAN) 3

N[1],*N*[2]-bis(4-methoxyphenyl)acenaphthylene-1,2-diimine (4-OMe-BIAN) 3

N[1],*N*[2]-bis(4-hexyloxyphenyl)acenaphthylene-1,2-diimine (4-*n*-hexyloxy-BIAN) 4

Synthesis of Ag(I) BIAN complexes 4

$[Ag(4\text{-methylthio-BIAN})_2]BF_4$ (Ag1) 4

$[Ag(4\text{-}n\text{-hexylthio-BIAN})_2]BF_4$ (Ag2) 4

$[Ag(4\text{-OMe-BIAN})_2]BF_4$ (Ag3). 5

$[Ag(4\text{-}n\text{-hexyloxy-BIAN})_2]BF_4$ (Ag4) 5

Spectroscopic data 6

Electrocatalysis data 21

Materials and Methods

All chemicals were purchased from Sigma-Aldrich, Strem, BLDpharm, TCI or Merck and used without further purification unless stated otherwise. Anhydrous ACN was obtained from a molar sieve MB-SPS-7 (M. Braun Inertgas-Systeme GmbH) under argon atmosphere. The deuterated NMR solvents $CDCl_3$ and CD_3CN were obtained from Euriso-Top. Proton (^1H NMR) and carbon (^{13}C NMR) nuclear magnetic resonance spectra were recorded on a Bruker Advance 300 MHz NMR spectrometer and a Bruker DRX 500 MHz spectrometer equipped with a cryoprobe (TXI). The chemical shifts are given in parts per million (ppm) on the delta scale (δ) and referenced to the employed deuterated solvent for ^1H NMR and ^{13}C NMR spectra. UV-vis absorption spectra were collected on a Varian CARY 300 Bio spectrophotometer from 200 to 900 nm at ambient temperatures. High resolution mass spectra were obtained utilizing an Agilent 6520 Q-TOF mass spectrometer with an ESI source, an Agilent G1607A coaxial sprayer and ammonium formate containing eluents.

Synthesis procedures

Preparation of 4-*n*-hexylthioaniline

4-Amino-thiophenol (4.35 g, 33.7 mmol) was dissolved in 175 mL dry ACN in a flame-dried Schlenk flask, cooled in an ice-bath and KO*t*bu (4.73 g, 42.2 mmol, 1.25 eq.) added under argon. The resulting light pink suspension was stirred for 1 h while being allowed to warm to rt. Ensuing, 1-iodhexane (7.51 g, 35.4 mmol, 1.05 eq.) was added and the mixture stirred for further 21 h at rt under argon. Afterwards it was extracted with DCM (300 mL) and water (200 mL) with the addition of brine to enhance phase separation. The organic extract was washed with brine (200 mL), filtered over a silica plug, dried over Na_2SO_4 and concentrated *in vacuo* to afford a brown-yellow oil. Finally, the crude was purified *via* column chromatography (silica, gradient eluent *n*-heptane/DCM: 2:1 to DCM) and the yellow band collected to afford the desired product as yellow oil in 65% yield.

4-*n*-hexylthioaniline

^1H NMR (300 MHz, $CDCl_3$, 25 °C) δ / ppm: 0.87 (t, J = 6.8 Hz, 3 H, CH_3), 1.21 – 1.42 (m, 6 H, CH_2), 1.56 (p, J = 7.4 Hz, 2 H, CH_2), 2.76 (t, J = 6.8 Hz, 2 H, CH_2), 3.67 (bs, 2 H, NH_2), 6.62 (dt, J_1 = 8.6 Hz, J_2 = 2.8 Hz, 2 H, ArH), 7.23 (dt, J_1 = 8.6 Hz, J_2 = 2.8 Hz, 2 H, ArH)
^{13}C NMR (126 MHz, $CDCl_3$, 25 °C) δ / ppm: 14.2, 22.7, 28.5, 29.5, 31.5, 36.5, 115.7, 124.0, 133.8, 145.8
HRMS (ESI) *m*/*z* calcd. for $C_{36}H_{41}N_2S_2^+$: 210.1316 [*M*-H]$^+$; found: 210.1314.

General Procedure for the Preparation of BIAN Ligands

Acenaphthenequinone was combined with a substoichiometric amount of acetic acid (25 mol%) at room temperature, followed by addition of a slight excess of either 4-(methylthio)aniline, 4-(n-hexylthio)aniline, p-anisidine or 4-(n-hexyloxy)aniline. Milling was performed at 30 Hz in a 10 mL stainless steel grinding jar containing ten 2 mm stainless steel balls at room temperature for 30 min, affording the free BIAN ligands. The crude products were extracted with DCM and 0.1 M aqueous HCl. Following this, the organic phase was washed with deionized water, dried over Na_2SO_4, and concentrated under reduced pressure. The isolated yields of the ligands (**L1**–**L4**) were 89-94%.

***N¹*,*N²*-bis(4-methylthiophenyl)acenaphthylene-1,2-diimine (4-SMe-BIAN)**

^{1}H NMR (500 MHz, $CDCl_3$, 25 °C) δ / ppm: 2.56 (s, 6 H, CH_3), 7.01 (d, *J* = 7.3 Hz, 2 H, ArH), 7.09 (d, *J* = 8.5 Hz, 4 H, ArH), 7.37 – 7.42 (m, 6 H, ArH), 7.90 (d, *J* = 8.2 Hz, 2 H, ArH).

^{13}C NMR (126 MHz, $CDCl_3$, 25 °C) δ / ppm: 16.7, 119.3, 124.1, 127.8, 128.2, 128.6, 129.2, 131.4, 134.0, 141.9, 149.2, 161.6.

UV-vis (DCM) λ_{max} nm (log ε): 230 (4.82), 261 (4.59), 300 (4.09), 448 (3.58).

HRMS (ESI) *m*/*z* calcd. for $C_{26}H_{20}N_2S_2$+H^+: 425.1146 [*M*+H]$^+$; found: 425.1147.

***N¹*,*N²*-bis(4-hexylthiophenyl)acenaphthylene-1,2-diimine (4-*n*-hexylthio-BIAN)**

^{1}H NMR (300 MHz, $CDCl_3$, 25 °C) δ / ppm: 0.93 (t, *J* = 6.9 Hz, 6 H, CH_3), 1.28 – 1.42 (m, 8 H, CH_2), 1.50 (p, *J* = 7.4 Hz, 4 H, CH_2), 1.74 (p, *J* = 7.6 Hz, 4 H, CH_2), 3.01 (t, *J* = 7.4 Hz, 4 H, CH_2), 7.01 (d, *J* = 7.2 Hz, 2 H, ArH), 7.10 (dt, J_1 = 8.5 Hz, J_2 = 1.8 Hz, 4 H, ArH), 7.40 – 7.50 (m, 6 H, ArH), 7.94 (d, *J* = 8.2 Hz, 2 H, ArH).

^{13}C NMR (126 MHz, $CDCl_3$, 25 °C) δ / ppm: 14.2, 22.7, 28.7, 29.3, 31.6, 34.6, 119.2, 124.1, 127.8, 128.6, 129.3, 130.9, 131.4, 132.5, 141.9, 149.9, 161.5.

UV-vis (DCM) λ_{max} nm (log ε): 232 (4.79), 261 (4.56), 302 (4.02), 432 (3.63).

HRMS (ESI) *m*/*z* calcd. for $C_{36}H_{41}N_2S_2^+$: 565.2711 [*M*-H]$^+$; found: 565.2709.

***N¹*,*N²*-bis(4-methoxyphenyl)acenaphthylene-1,2-diimine (4-OMe-BIAN)**

^{1}H NMR (300 MHz, $CDCl_3$, 25 °C) δ / ppm: 3.90 (s, 6 H, CH3), 7.00 – 7.04 (m, 6 H, ArH), 7.08 – 7.11 (m, 4 H, ArH), 7.39 (dd, J_1 = 7.3 Hz, J_2 = 8.2 Hz, 2 H, ArH), 7.89 (d, *J* = 8.2 Hz, 2 H, ArH).

^{13}C NMR (75 MHz, $CDCl_3$, 25 °C) δ / ppm: 55.6, 114.8, 119.9, 123.8, 127.7, 128.9, 129.0, 131.4, 145.1, 157.0, 161.7.

UV-vis (ACN) $\lambda_{max\ nm}$ (log ε): 212 (4.64), 229 (4.83), 291 (4.08), 424 (3.63).

UV-vis (DCM) $\lambda_{max\ nm}$ (log ε): 232 (4.95), 293 (4.19), 431 (3.72).

IR (25 °C) *ν* / cm^{-1}: 3043, 3033, 3003, 2972, 2949, 2939, 2913, 2840, 1642, 1599, 1579, 1499, 1484, 1463, 1441, 1434, 1414, 1286, 1272, 1231, 1178, 1165, 1108, 1088, 1028, 926, 833, 811, 786, 743, 718, 586, 544, 529, 502, 467, 418.

N^1,N^2-bis(4-hexyloxyphenyl)acenaphthylene-1,2-diimine (4-*n*-hexyloxy-BIAN)

^{1}H NMR (500 MHz, $CDCl_3$, 25 °C) δ / ppm: 0.92 – 0.95 (m, 6 H, CH3), 1.36 – 1.41 (m, 8 H, CH2), 1.49 – 1.55 (m, 4 H, CH2), 1.81 – 1.87 (m, 4 H, CH2), 4.03 (t, *J* = 6.6 Hz, 4 H, CH2), 6.99 – 7.02 (m, 4 H, ArH), 7.04 – 7.09 (m, 6 H, ArH), 7.39 (t, *J* = 7.8 Hz, 2 H, ArH), 7.88 (d, *J* = 8.2 Hz, 2 H, ArH).
^{13}C NMR (126 MHz, $CDCl_3$, 25 °C) δ / ppm: 14.2, 22.8, 26.0, 29.5, 31.8, 68.5, 115.3, 119.9, 123.8, 127.7, 128.9, 128.9, 131.4, 141.8, 144.9, 156.6, 161.7.
UV-vis (ACN) $\lambda_{max\ nm}$ (log *ε*): 230 (5.09), 290 (4.44), 422 (3.92).
UV-vis (DCM) $\lambda_{max\ nm}$ (log *ε*): 232 (4.96), 292 (4.20), 434 (3.75).
IR (25 °C) *ν* / cm^{-1}: 3055, 2930, 2894, 2863, 1657, 1638, 1624, 1601, 1591, 1498, 1473, 1455, 1435, 1418, 1389, 1362, 1285, 1274, 1237, 1183, 1167, 1148, 1127, 1107, 1092, 1046, 1025, 996, 973, 933, 921, 901, 832, 805, 784, 753, 741, 656, 640, 599, 538, 522, 516, 486, 465, 444, 434, 422, 414.
HRMS (ESI) *m*/*z* calcd for $C_{36}H_{40}N_2O_2$+H^+: 533.3163 $[M+H]^+$; found: 533.3160.

Synthesis of Ag(I) BIAN complexes

Homoleptic Ag(I) BIAN complexes were prepared also via ball-milling procedure. $AgBF_4$ (0.2 mmol) was mixed with BIAN ligand (0.4 mmol, 2 eq.) leading to immediate color changes. The mixtures were subsequently ground at room temperature in the dark for 15 min, and yielded the respective pure homoleptic Ag(I) bis-BIAN complexes **Ag1**-**Ag4**.

[Ag(4-methylthio-BIAN)$_2$]BF_4 (Ag1)

^{1}H NMR (500 MHz, CD_3CN, 25 °C) δ / ppm: 2.50 (s, 12 H, CH_3), 6.91 (d, *J* = 8.6 Hz, 8 H, ArH), 7.11 (d, *J* = 7.3 Hz, 4 H, ArH), 7.20 (d, *J* = 8.6 Hz, 8 H, ArH), 7.52 (t, *J* = 7.7 Hz, 4 H, ArH), 8.11 (d, *J* = 8.2 Hz, 4 H, ArH)
^{13}C NMR (126 MHz, CD_3CN, 25 °C) δ / ppm: 15.3, 120.2, 124.6, 127.6, 128.3, 130.7, 131.3, 136.3, 142.0, 146.6, 160.8
UV-vis (ACN) λ_{max} nm (log *ε*): 203 (5.11), 229 (5.14), 259 (4.99), 300 (4.43), 429 (3.98)
HRMS (ESI) *m*/*z* calcd. for $C_{52}H_{40}AgN_4S_4^+$: 955.1187 $[M-BF_4]^+$; found: 955.1185

[Ag(4-*n*-hexylthio-BIAN)$_2$]BF_4 (Ag2)

^{1}H NMR (500 MHz, $CDCl_3$, 25 °C) δ / ppm: 0.89 (t, *J* = 6.9 Hz, 12 H, CH_3), 1.30 – 1.34 (m, 16 H, CH_2), 1.48 (p, *J* = 7.4 Hz, 8 H, CH_2), 1.71 (p, *J* = 7.5 Hz, 8 H, CH_2), 3.03 (t, *J* = 7.4 Hz, 8 H, CH_2), 7.01 (d, *J*

= 8.3 Hz, 8 H, ArH), 7.10 (d, *J* = 7.3 Hz, 4 H, ArH), 7.35 (d, *J* = 8.4 Hz, 8 H, ArH), 7.46 (t, *J* = 7.8 Hz, 4 H, ArH), 8.03 (d, *J* = 8.3 Hz, 2 H, ArH)

^{13}C NMR (126 MHz, $CDCl_3$, 25 °C) δ / ppm: 14.2, 22.7, 28.7, 29.1, 31.6, 34.2, 120.4, 125.2, 127.2, 128.3, 130.0, 131.0, 131.2, 135.0, 142.6, 147.2, 161.6

UV-vis (DCM) λ_{max} nm (log ε): 233 (5.14), 266 (5.03), 441 (4.10)

HRMS (ESI) *m*/*z* calcd. for $C_{72}H_{80}N_4S_4Ag^+$: 1235.4316 [*M*-BF_4]$^+$; found: 1235.4315.

[Ag(4-OMe-BIAN)$_2$]BF_4 (Ag3).

^{1}H NMR (500 MHz, $CDCl_3$, 25 °C) δ / ppm: 3.90 (s, 12 H, CH3), 7.04 (d, *J* = 8.9 Hz, 8 H, ArH), 7.18 (d, *J* = 8.9 Hz, 8 H, ArH), 7.27 (d, overlapping, *J* = 8.0 Hz, 4 H, ArH) 7.50 (t, *J* = 8.0 Hz, 8 H, ArH), 8.03 (d, *J* = 8.2 Hz, 4 H, ArH).

^{13}C NMR (126 MHz, $CDCl_3$, 25 °C) δ / ppm: 55.9, 115.2, 121.6, 125.0, 127.2, 128.3, 131.0, 131.2, 141.8, 142.5, 158.7, 161.4.

UV-vis (ACN) $\lambda_{max\ nm}$ (log ε): 212 (4.92), 229 (5.12), 292 (4.36), 422 (3.88).

UV-vis (DCM) $\lambda_{max\ nm}$ (log ε): 231 (5.25), 309 (4.51), 396 (4.13).

HRMS (ESI) *m*/*z* calcd for $C_{52}H_{40}AgN_4O_4^+$: 891.2100 [*M*-BF4]$^+$; found: 891.2096.

[Ag(4-*n*-hexyloxy-BIAN)$_2$]BF_4 (Ag4)

^{1}H NMR (500 MHz, CDCl3, 25 °C) δ / ppm: 0.94 – 0.95 (m, 12 H, CH3), 1.37 – 1.38 (m, 16 H, CH2), 1.48 – 1.54 (m, 8 H, CH2), 1.80 – 1.85 (m, 8 H, CH2), 4.02 (t, *J* = 6.4 Hz, 8 H, CH2), 7.01 (d, *J* = 8.4 Hz, 8 H, ArH), 7.13 (d, *J* = 8.4 Hz, 8 H, ArH), 7.27 (d, overlapping, *J* = 7.5 Hz, 4 H, ArH), 7.50 (t, *J* = 7.8 Hz, 4 H, ArH), 8.04 (d, *J* = 8.2 Hz, 4 H, ArH).

^{13}C NMR (126 MHz, CDCl3, 25 °C) δ / ppm: 14.2, 22.8, 25.9, 29.4, 31.8, 68.7, 115.3, 121.3, 125.0, 127.1, 128.3, 131.1, 131.2, 141.7, 142.5, 158.4, 161.4.

UV-vis (ACN) $\lambda_{max\ nm}$ (log ε): 230 (5.25), 290 (4.62), 420 (4.09).

UV-vis (DCM) $\lambda_{max\ nm}$ (log ε): 231 (5.21), 308 (4.45), 405 (4.08).

HRMS (ESI) *m*/*z* calcd for $C_{72}H_{80}AgN_4O_4^+$: 1172.5303 [*M*-BF_4]$^+$; found: 1172.5306.

Spectroscopic data

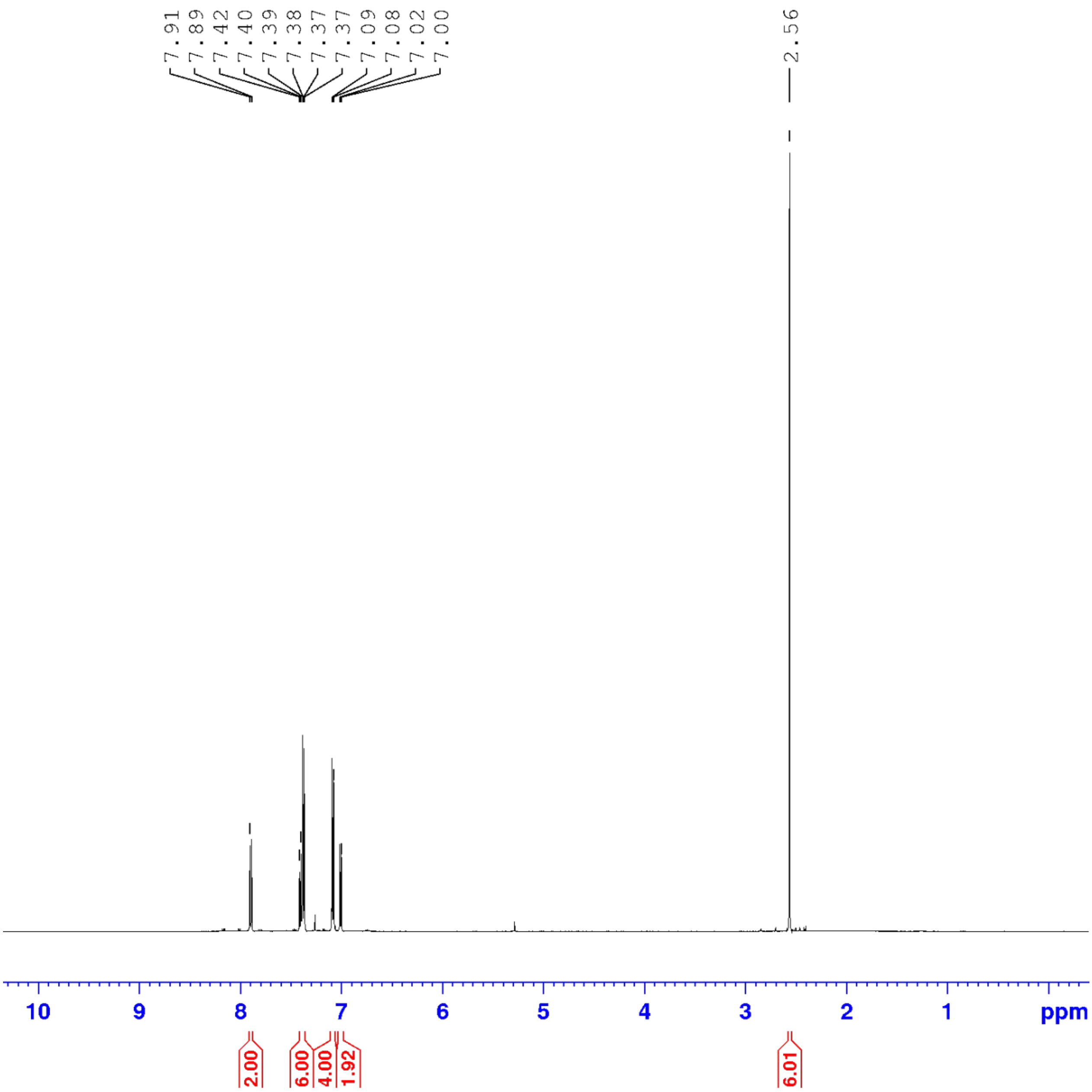

Figure S1. ^{1}H NMR (500 MHz, $CDCl_3$, 25 °C) of N^1,N^2-bis(4-(methylthio)phenyl)acenaphthylene-1,2-diimine **L1**.

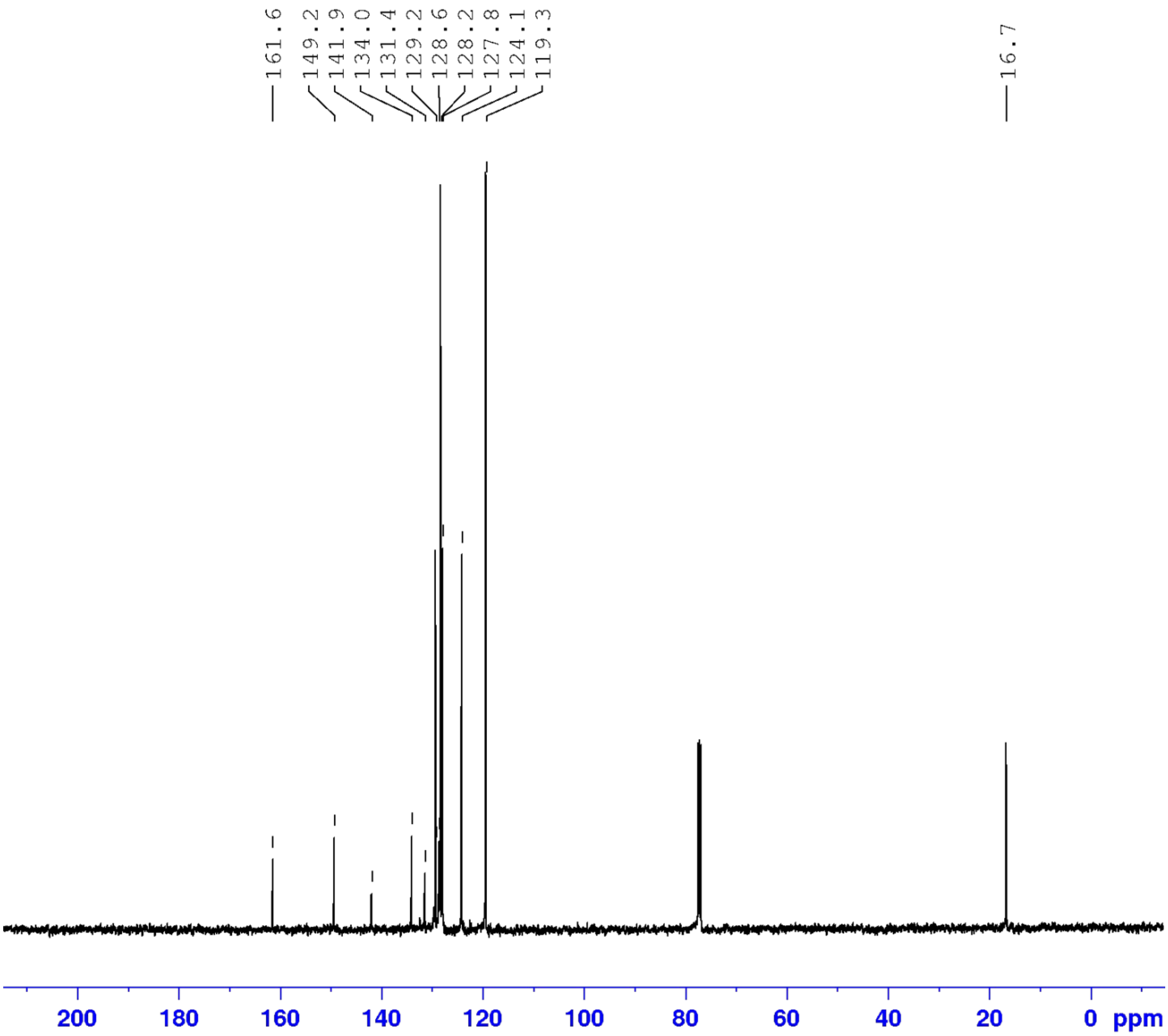

Figure S2. ^{13}C NMR (126 MHz, $CDCl_3$, 25 °C) of *N*[1],*N*[2]-bis(4-(methylthio)phenyl)acenaphthylene-1,2-diimine **L1**.

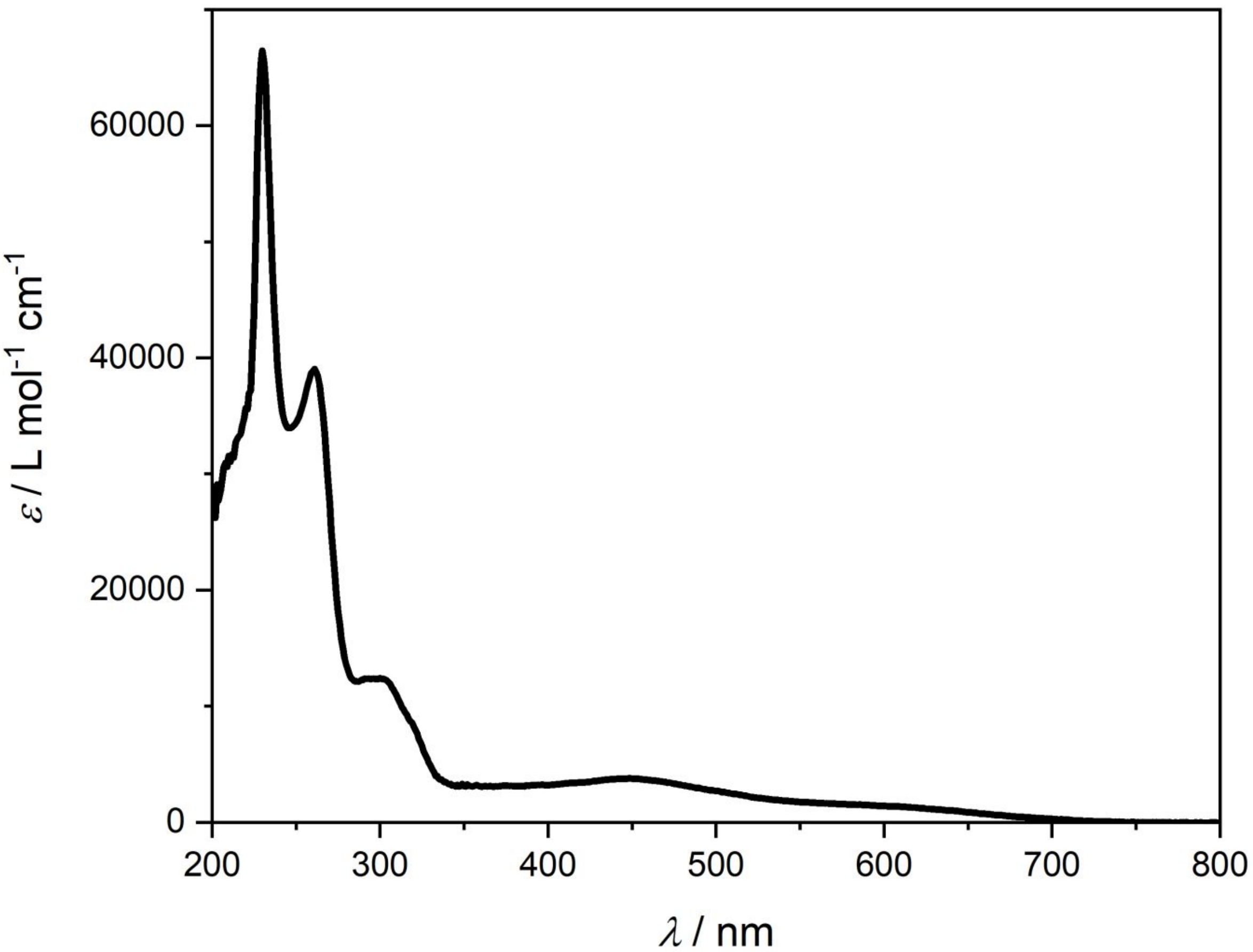

Figure S3. UV-vis absorption spectrum of N^1,N^2-bis(4-(methylthio)phenyl)acenaphthylene-1,2-diimine **L1** in DCM.

UV-vis (DCM) λ_{max} nm (log ε): 230 (4.82), 261 (4.59), 300 (4.09), 448 (3.58)

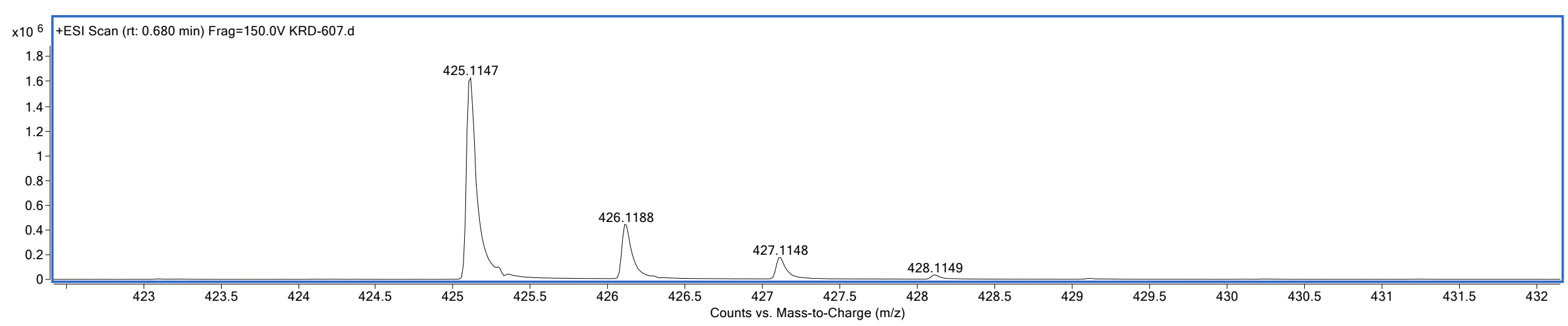

Figure S4. HRMS spectrum of N^1,N^2-bis(4-(methylthio)phenyl)acenaphthylene-1,2-diimine **L1**.

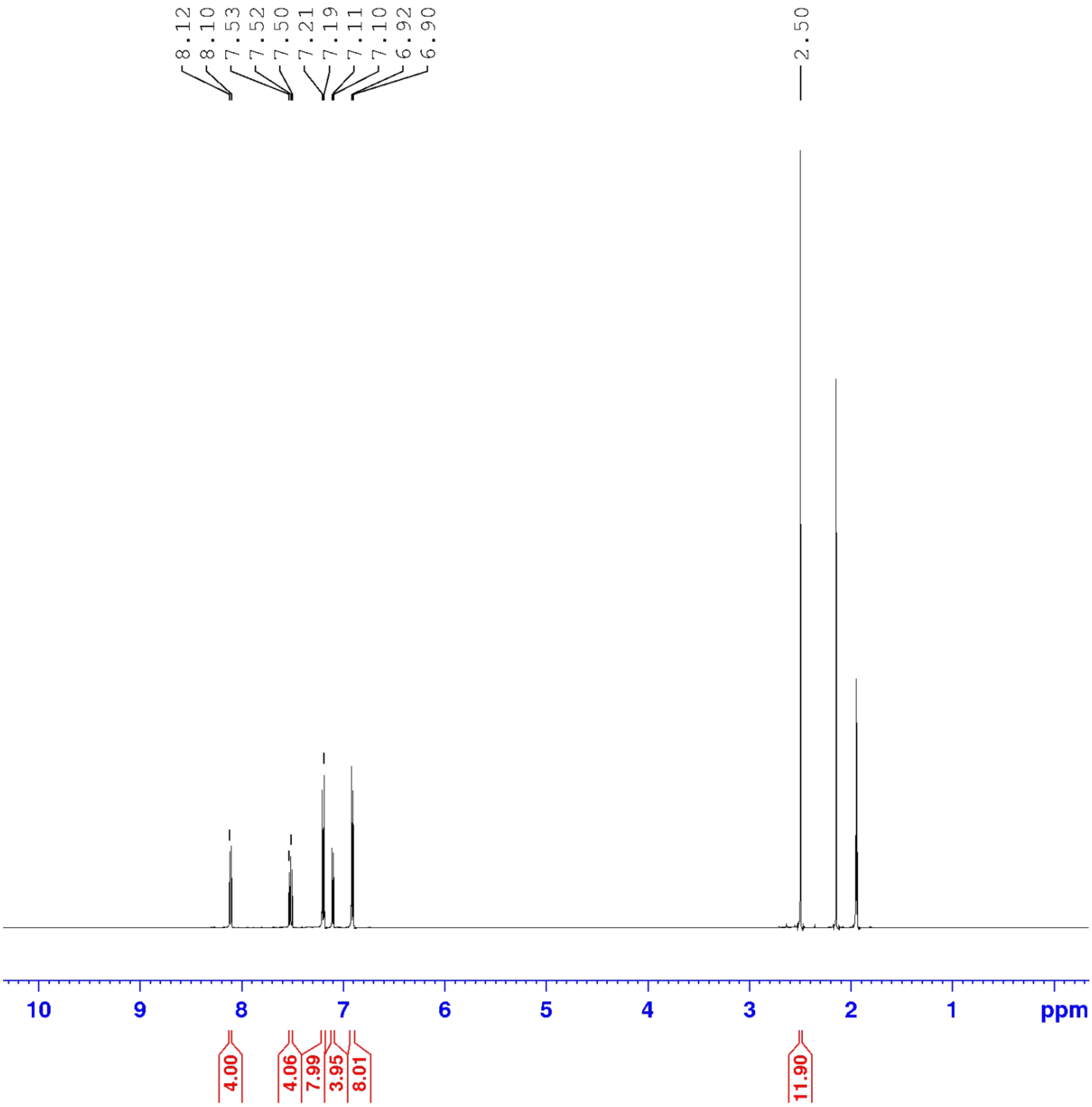

Figure S5. ^{1}H NMR (500 MHz, CD_3CN, 25 °C) of [Ag(4-SMe-BIAN)$_2$]BF_4 **Ag1**.

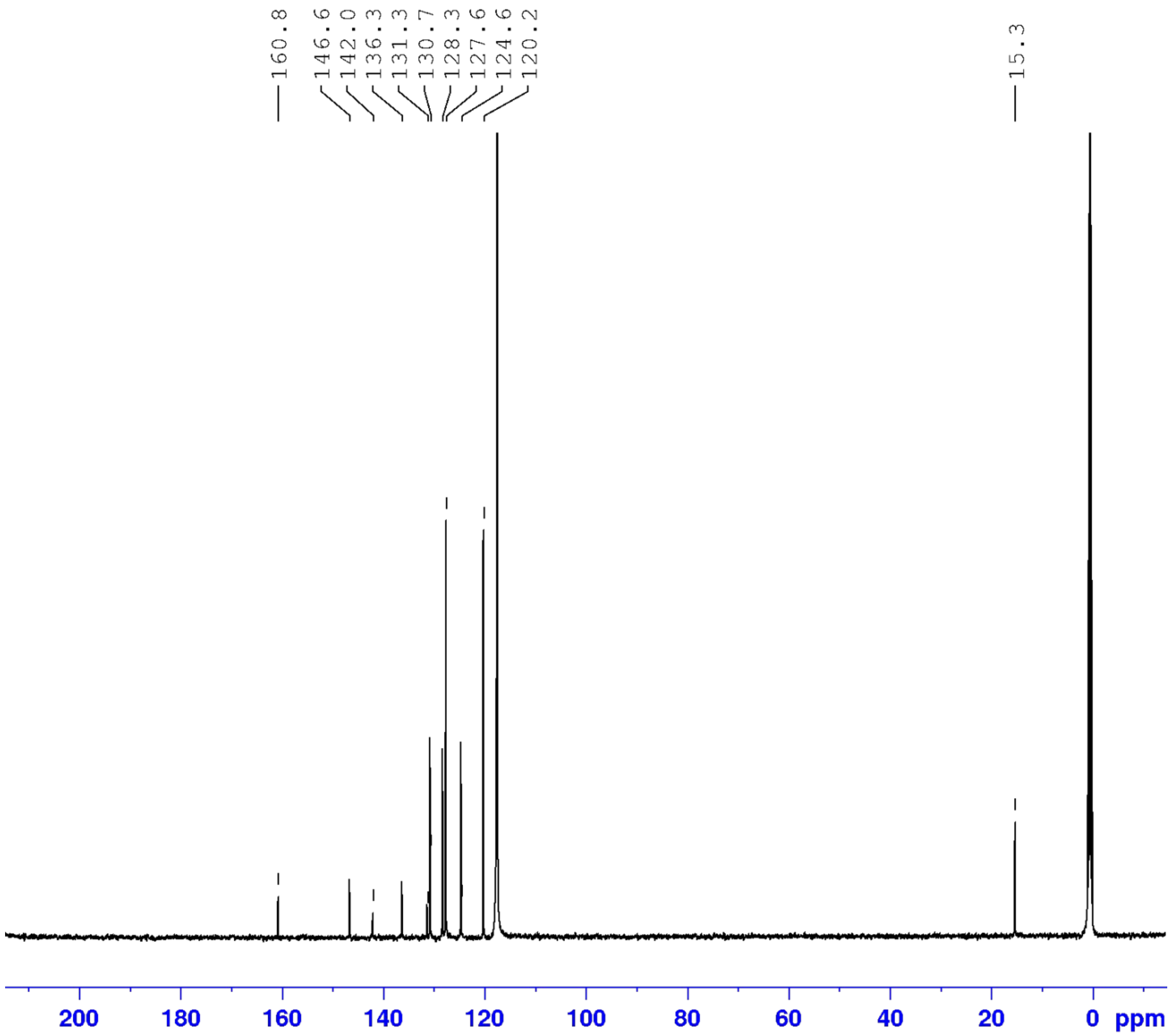

Figure S6. ^{13}C NMR (126 MHz, CD_3CN, 25 °C) of $[Ag(4\text{-SMe-BIAN})_2]BF_4$ **Ag1**.

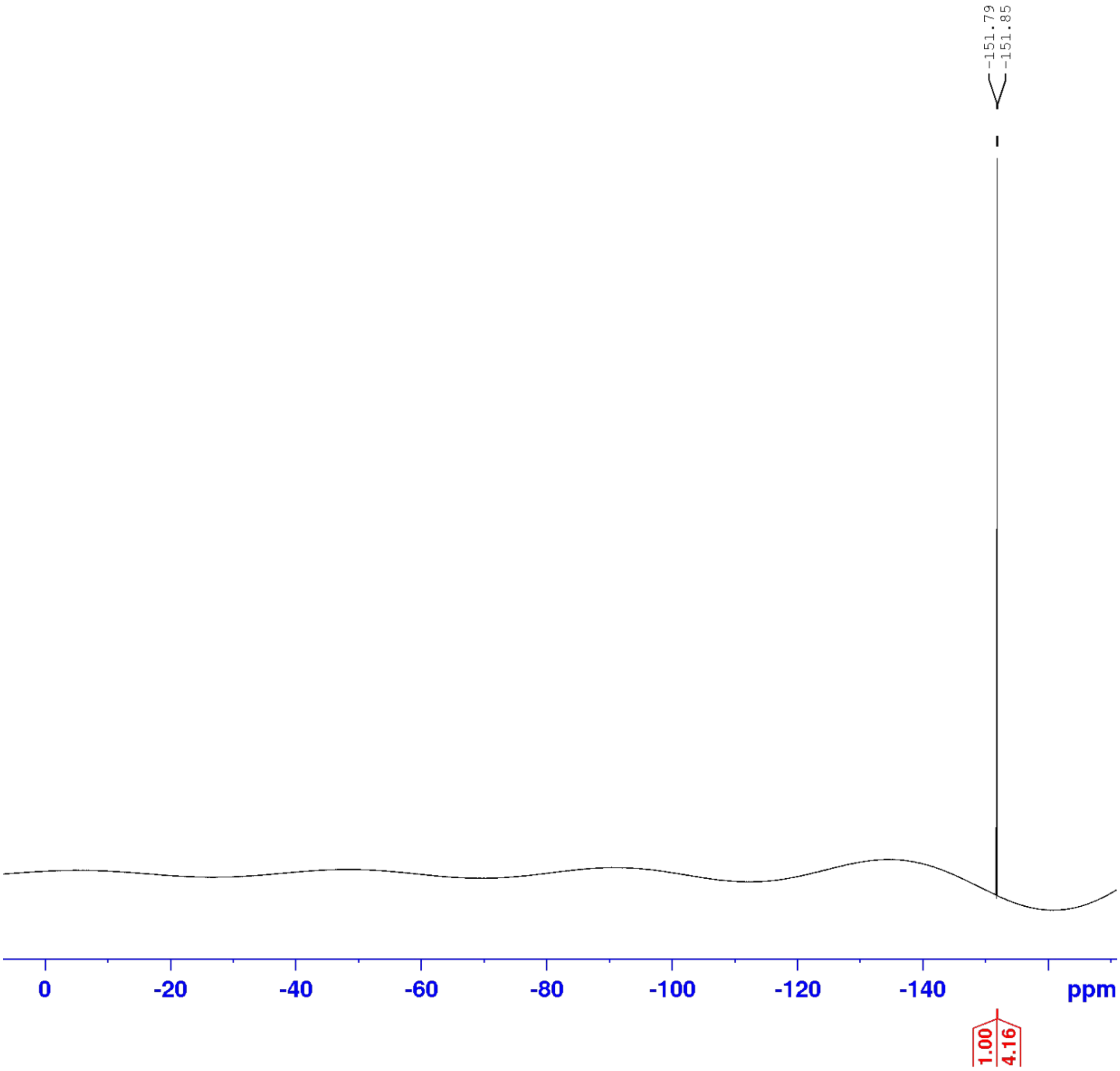

Figure S7. ^{19}F NMR (471 MHz, CD_3CN, 25 °C) of $[Ag(4\text{-}SMe\text{-}BIAN)_2]BF_4$ **Ag1**.

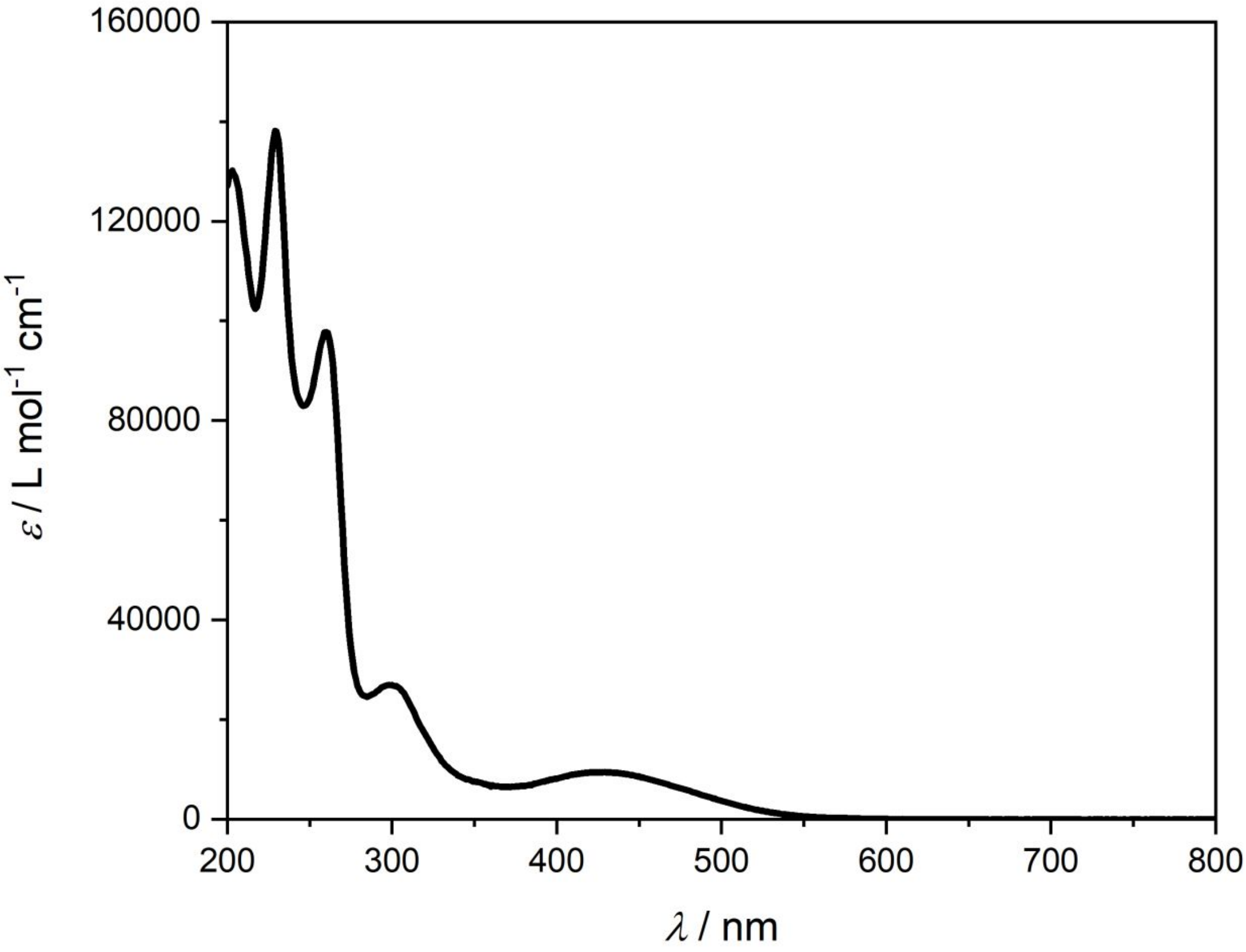

Figure S8. UV-vis absorption spectrum of $[Ag(4\text{-}SMe\text{-}BIAN)_2]BF_4$ **Ag1** in ACN.

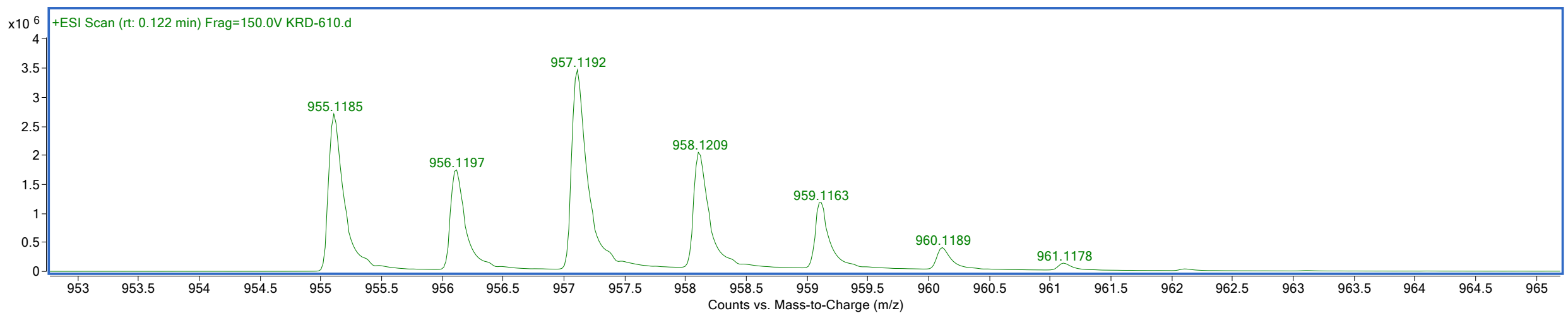

Figure S9. HRMS spectrum of $[Ag(4\text{-}SMe\text{-}BIAN)_2]BF_4$ **Ag1**.

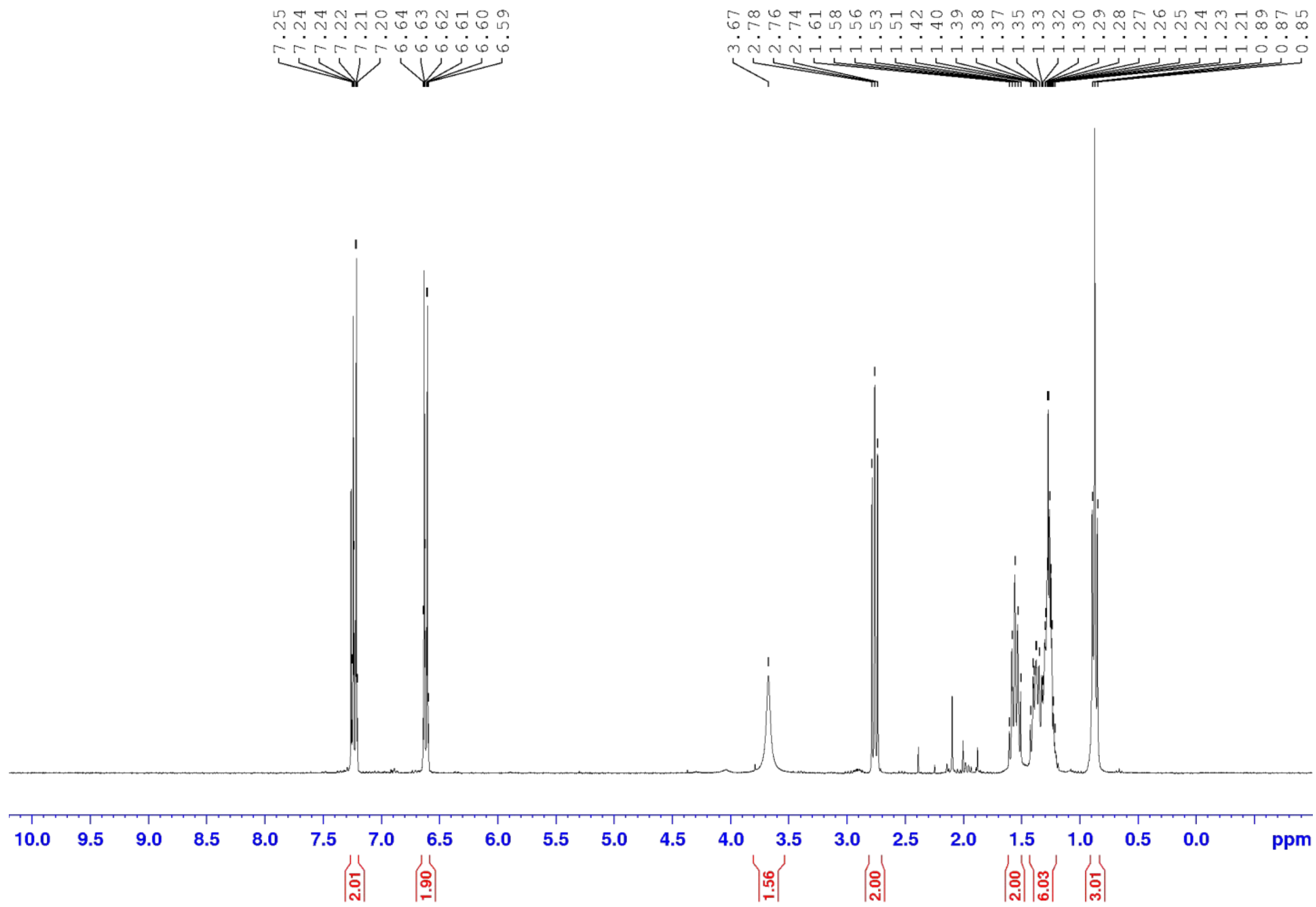

Figure S10. ^{1}H NMR (300 MHz, $CDCl_3$, 25 °C) of 4-(*n*-hexylthio)aniline.

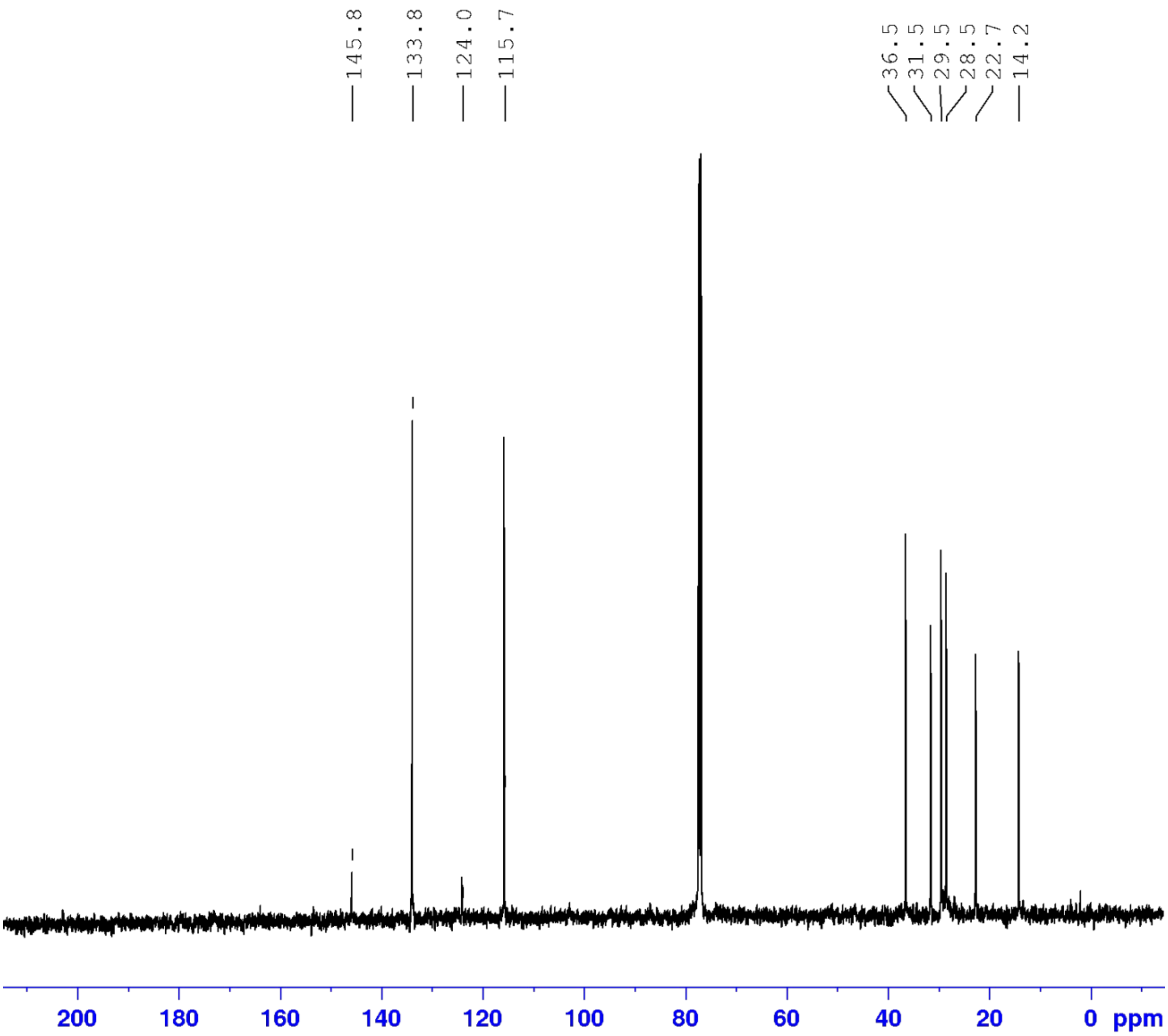

Figure S11. ^{13}C NMR (126 MHz, $CDCl_3$, 25 °C) of 4-(*n*-hexylthio)aniline.

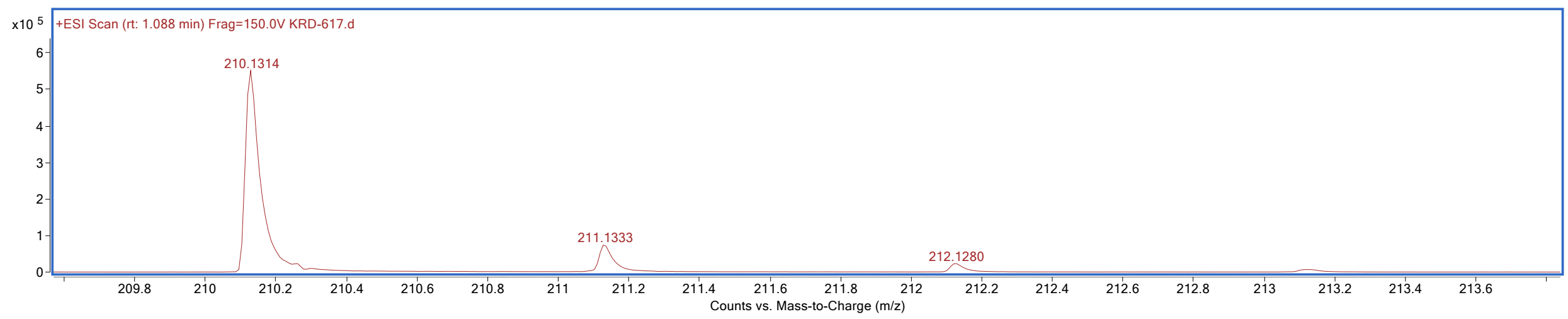

Figure S12. HRMS spectrum of 4-(*n*-hexylthio)aniline.

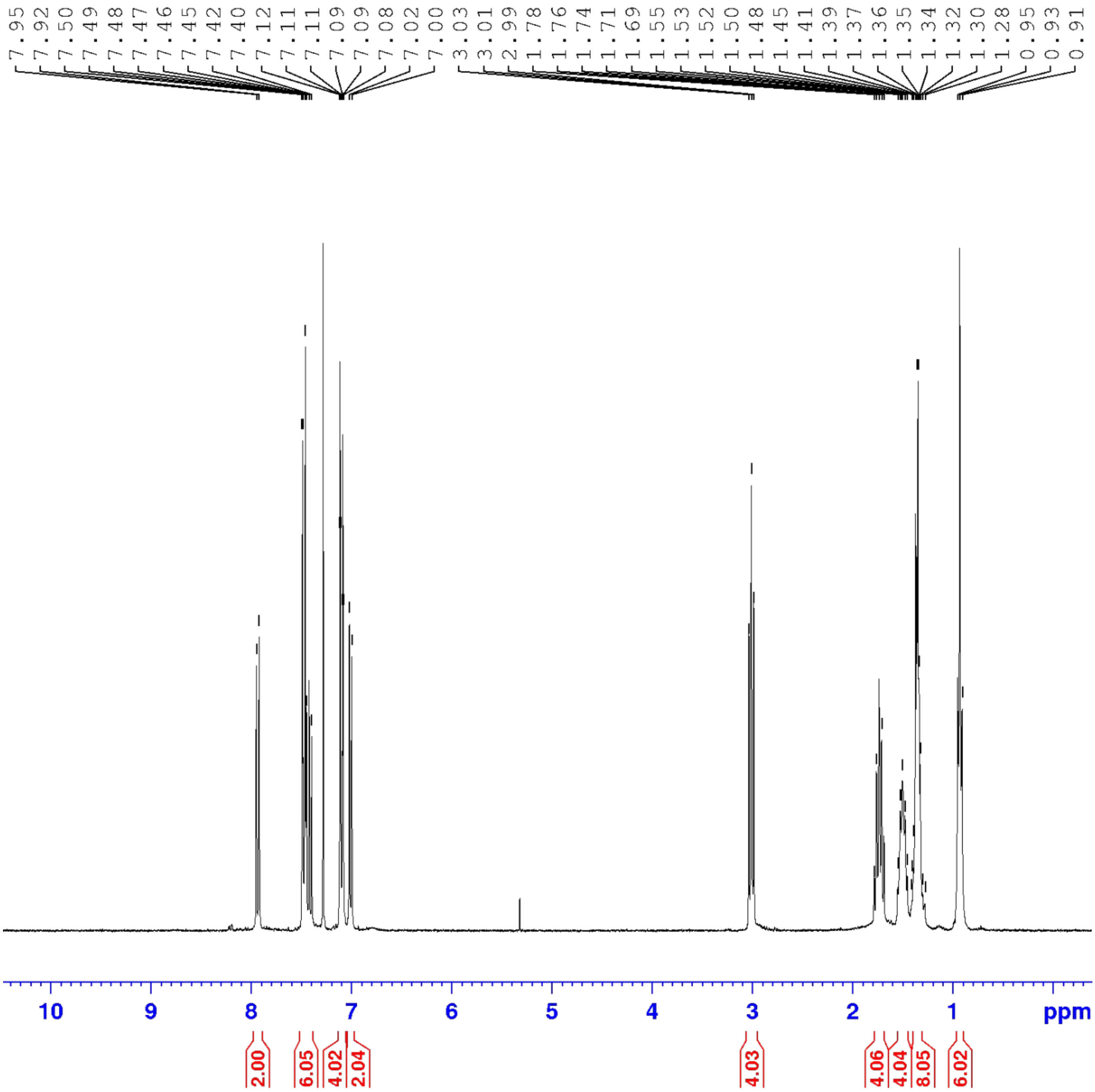

Figure S13. ^{1}H NMR (300 MHz, $CDCl_3$, 25 °C) of *N*[1],*N*[2]-bis(4-(*n*-hexylthio)phenyl)acenaphthylene-1,2-diimine **L2**.

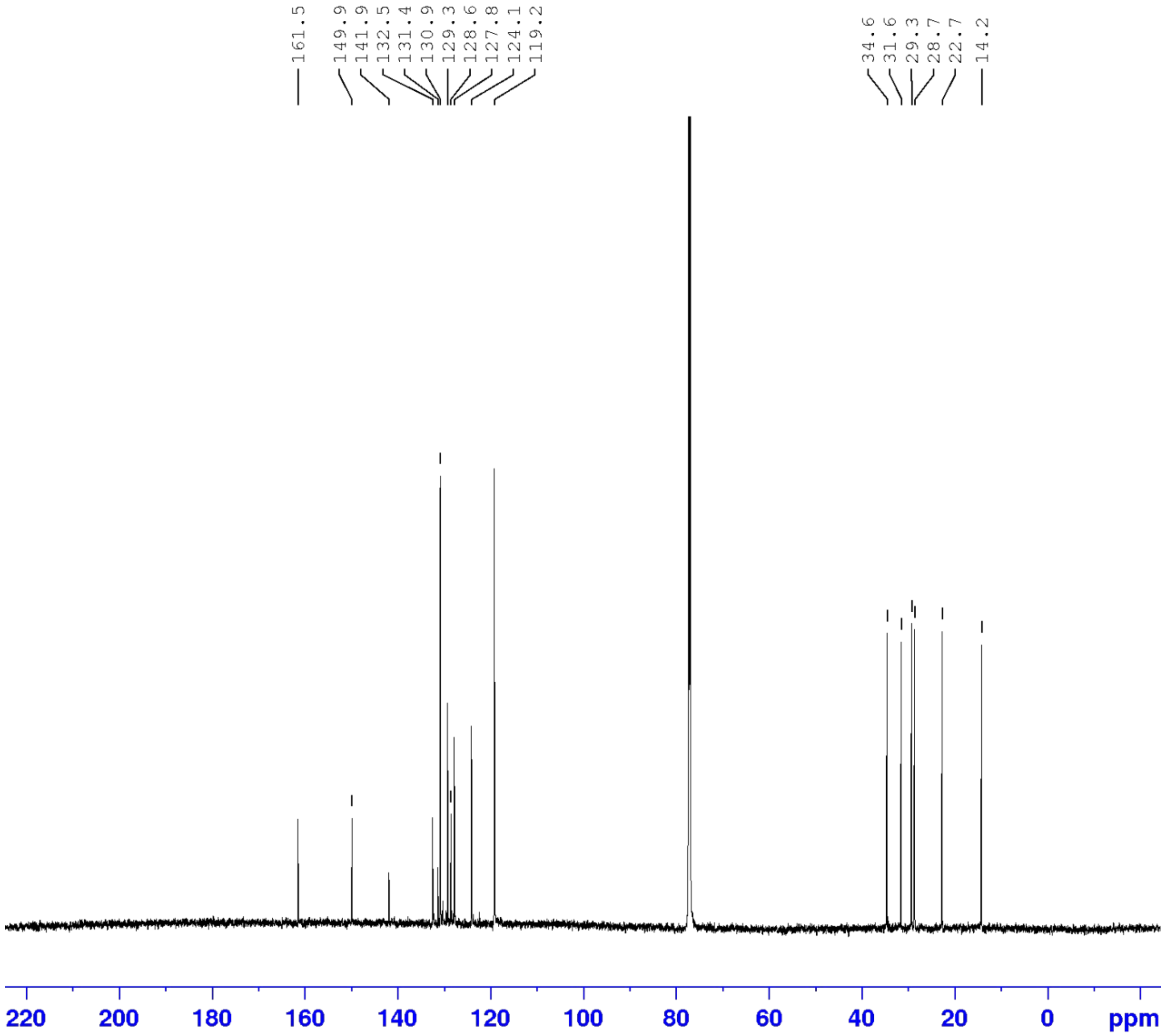

Figure S14. ^{13}C NMR (126 MHz, $CDCl_3$, 25 °C) of *N*[1],*N*[2]-bis(4-(*n*-hexylthio)phenyl)acenaphthylene-1,2-diimine **L2**.

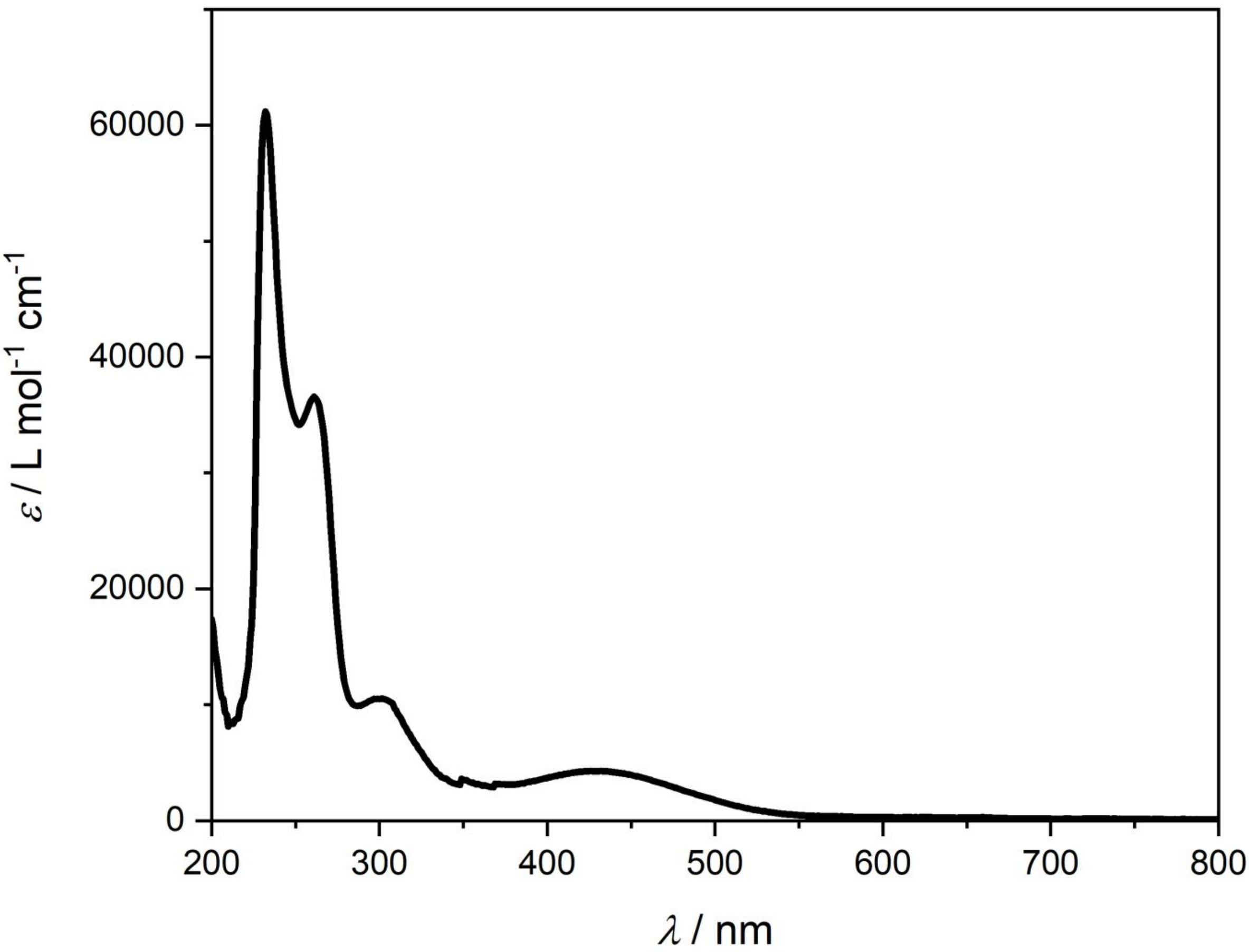

Figure S15. UV-vis absorption spectrum of N^1,N^2-bis(4-(hexylthio)phenyl)acenaphthylene-1,2-diimine **L2** in DCM.

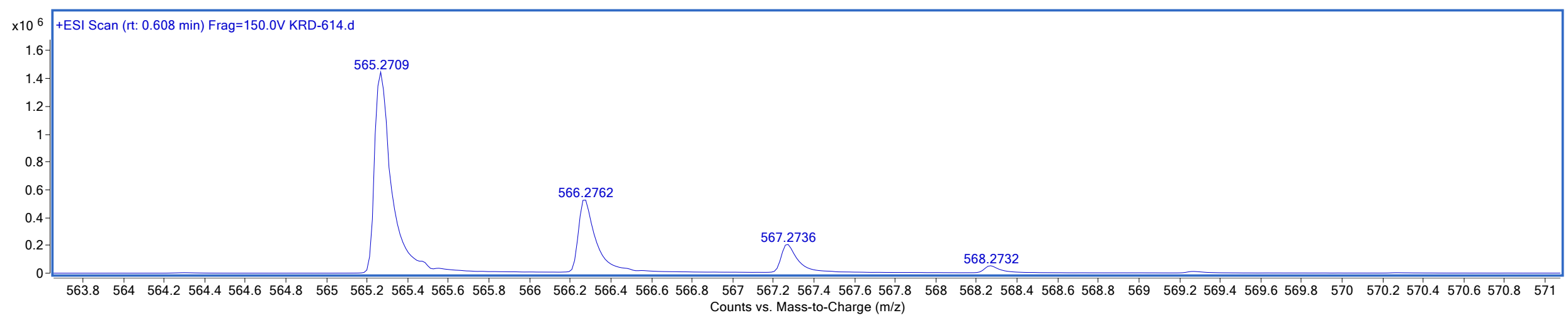

Figure S16. HRMS spectrum of N^1,N^2-bis(4-(hexylthio)phenyl)acenaphthylene-1,2-diimine **L2**.

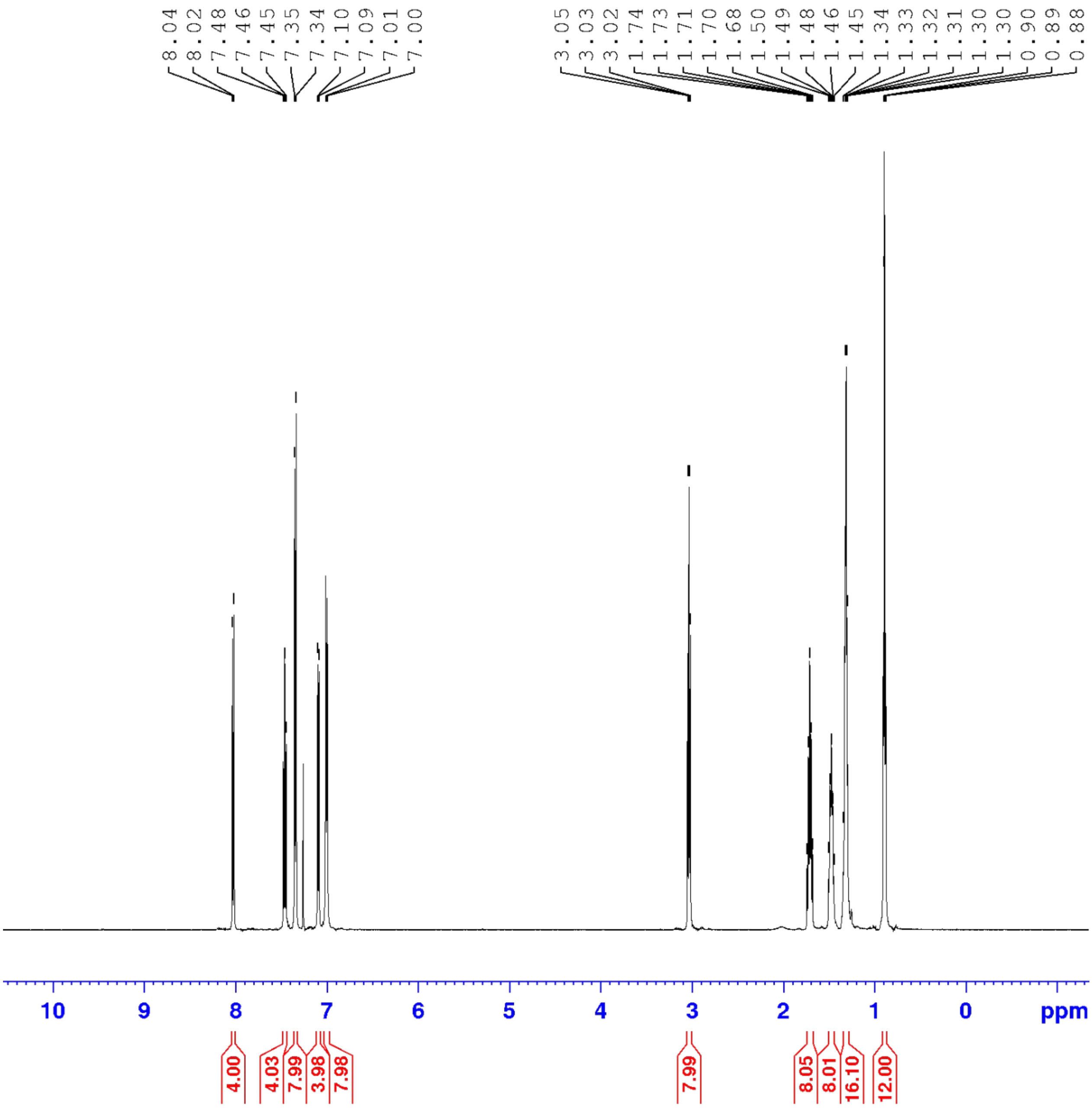

Figure S17. ^{1}H NMR (500 MHz, $CDCl_3$, 25 °C) of $[Ag(4\text{-}SC_6\text{-}BIAN)_2]BF_4$ **Ag2**.

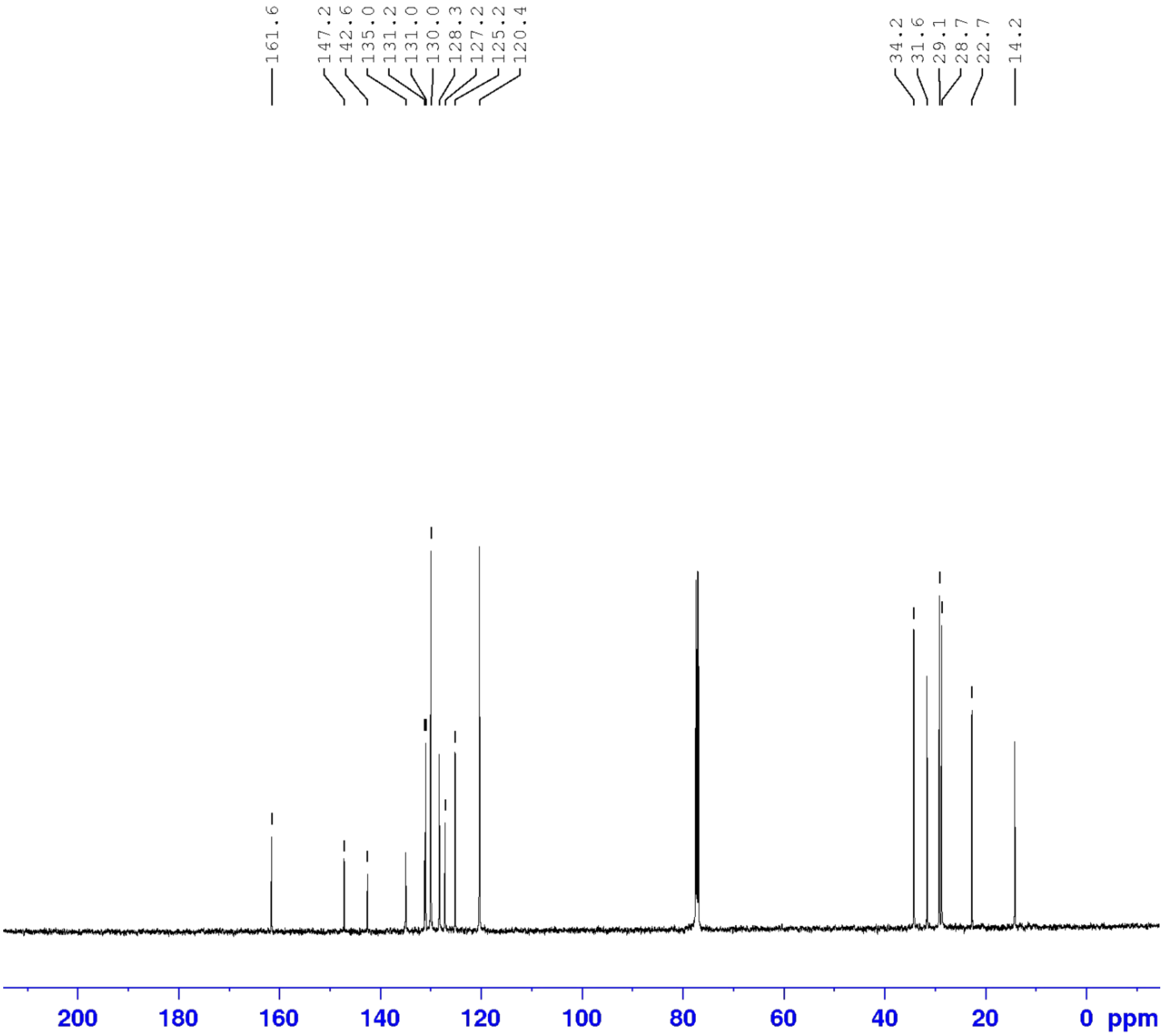

Figure S18. ^{13}C NMR (126 MHz, $CDCl_3$, 25 °C) of $[Ag(4\text{-}SC_6\text{-}BIAN)_2]BF_4$ **Ag2**.

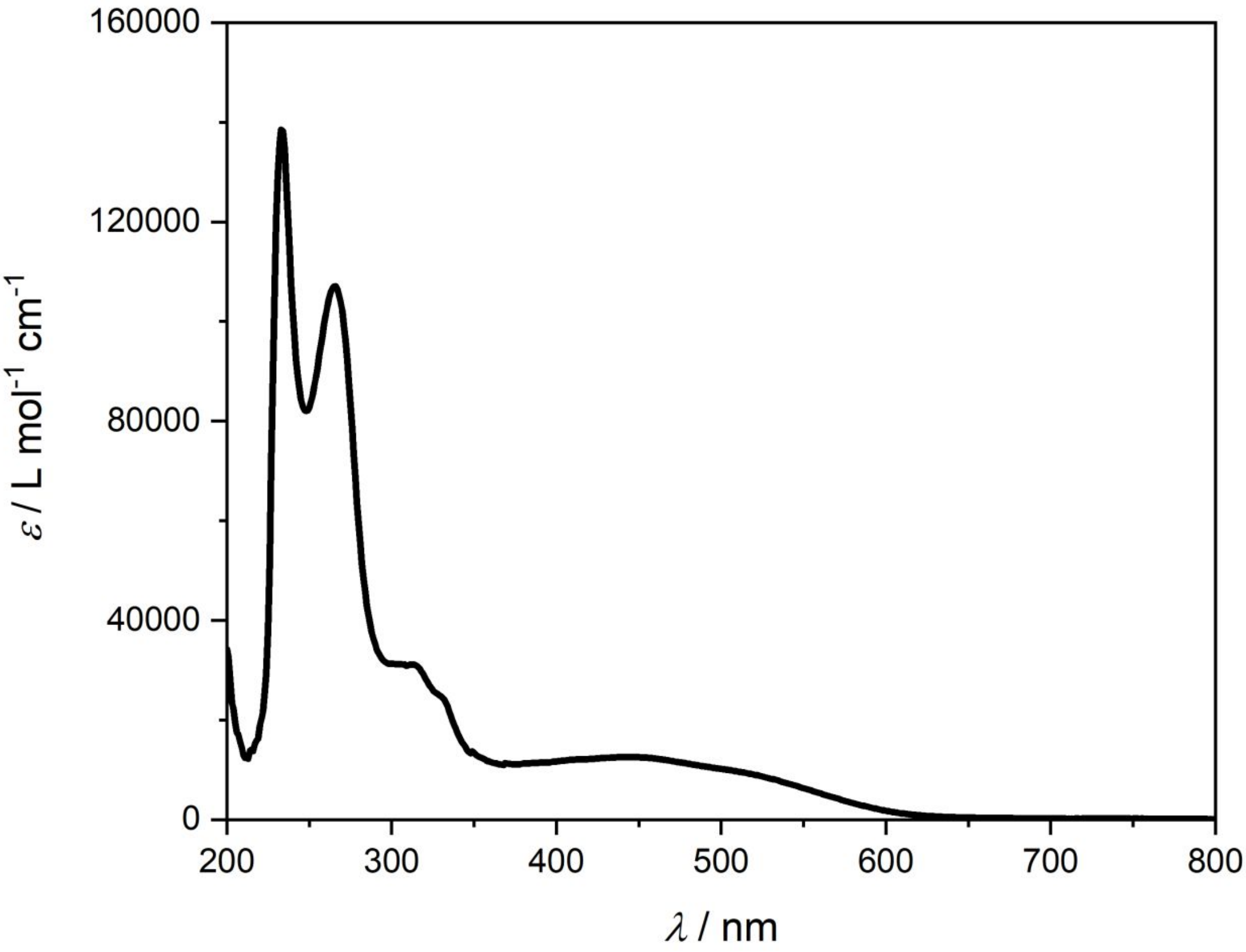

Figure S19. UV-vis absorption spectrum of $[Ag(4\text{-}SC_6\text{-}BIAN)_2]BF_4$ **Ag2** in DCM.

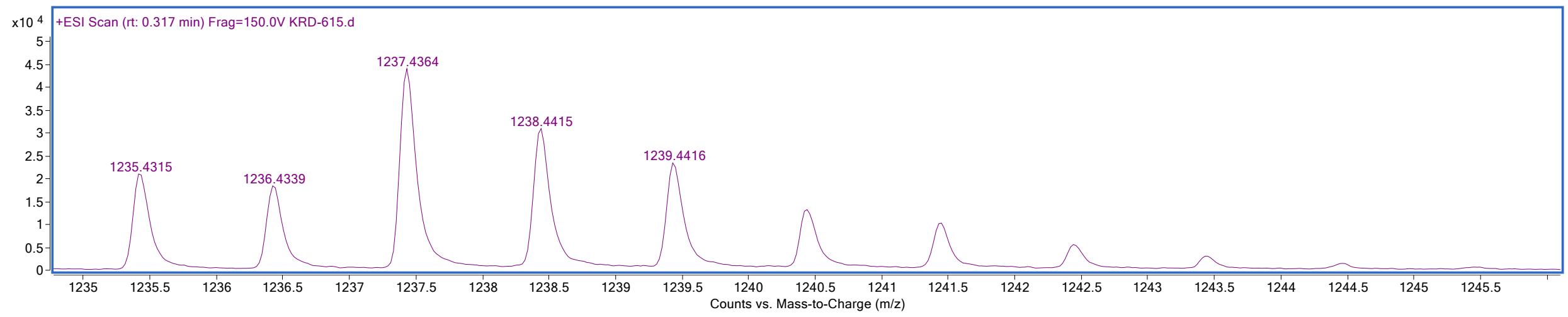

Figure S20. HRMS spectrum of $[Ag(4\text{-}SC_6\text{-}BIAN)_2]BF_4$ **Ag2**.

Electrocatalysis data

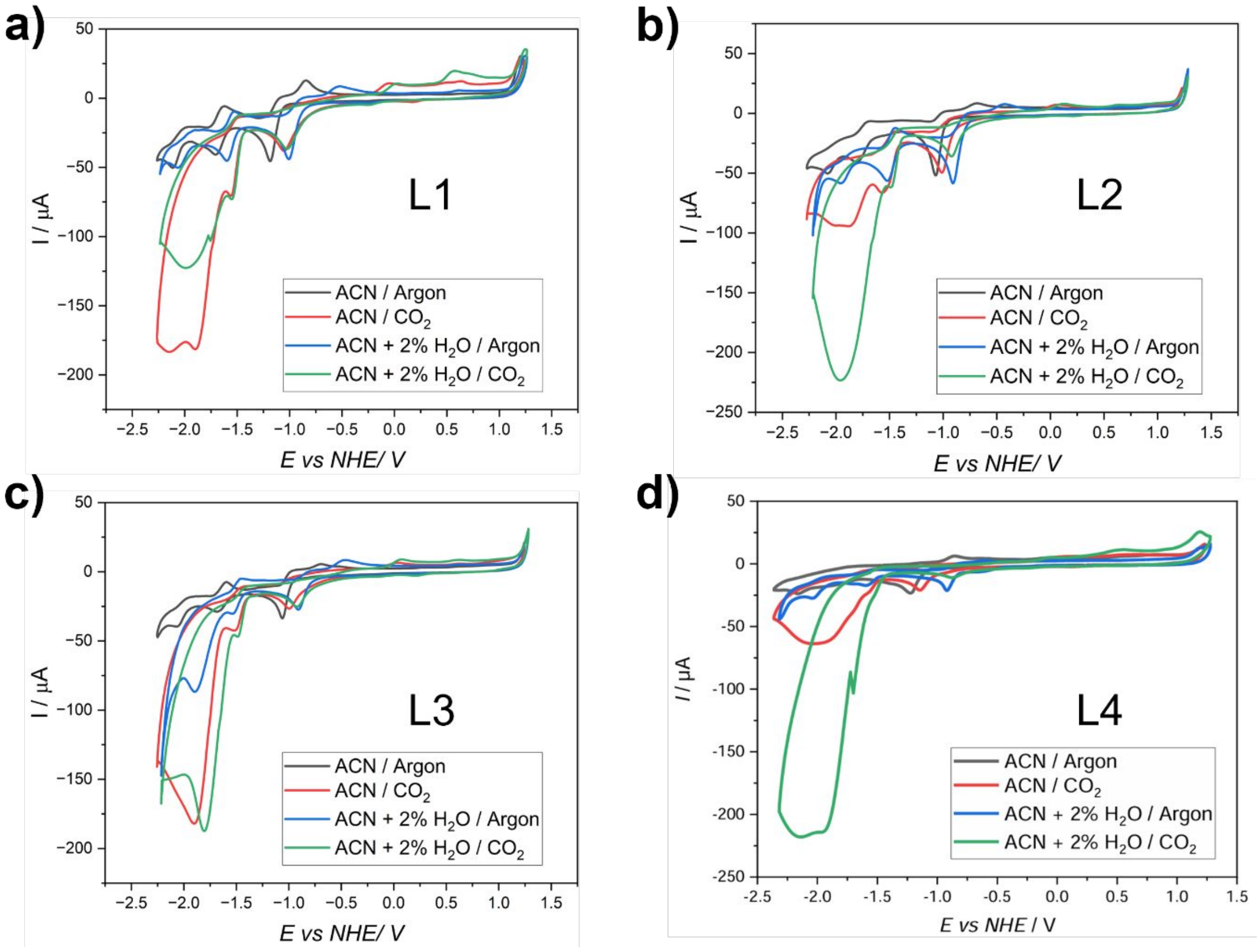

Figure S21. Cyclic voltammograms curves of bis-BIAN ligand a) **L1** (4-SMe-BIAN), b) **L2** (4-SC$_6$-BIAN), c) **L3** (4-OMe-BIAN) and d) **L4** (4-OC$_6$-BIAN) recorded in ACN with 0.1 M TBAPF$_6$ as the supporting electrolyte at scan rate of 100 mV s^{-1} under ACN/Ar (grey), ACN/CO_2 (red), ACN + 2% H_2O/Ar (blue), and ACN + 2% H_2O/CO_2 (green). All potentials are referenced to NHE via Fc/Fc$^+$ internal standard.

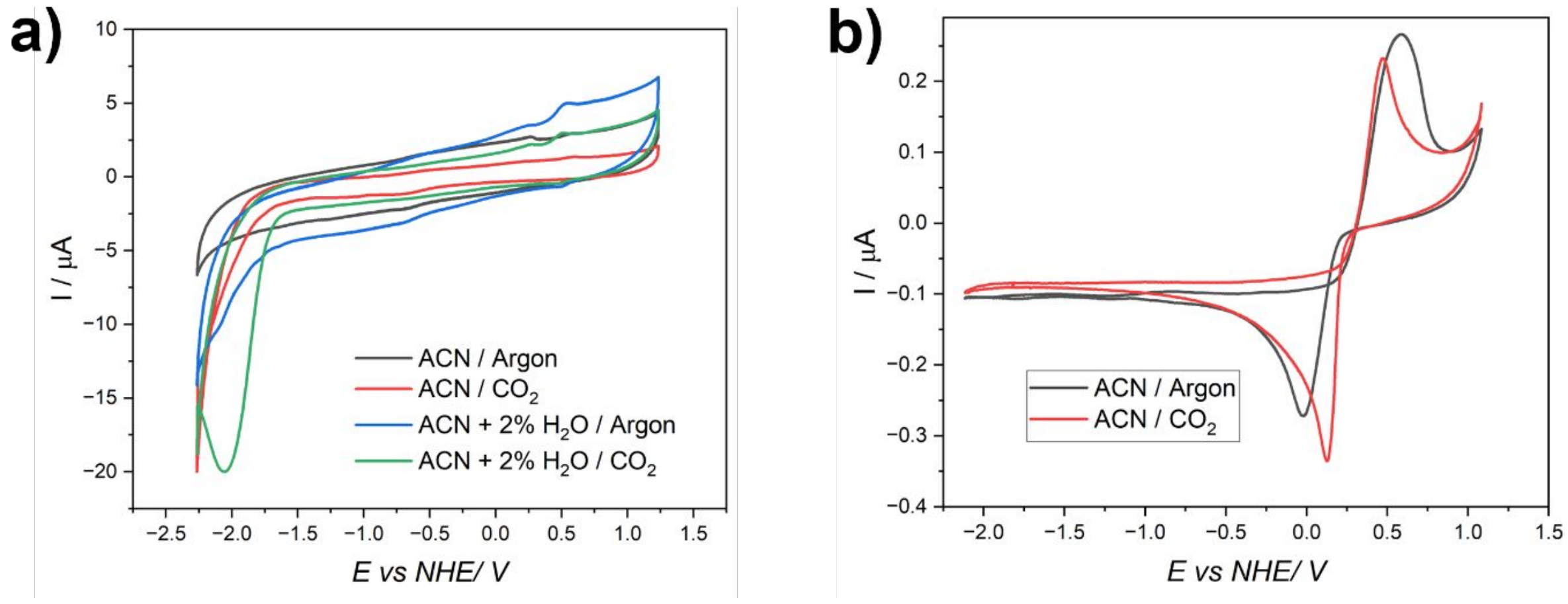

Figure S22. a) Cyclic voltammograms recorded in ACN with 0.1 M $TBAPF_6$ as the supporting electrolyte without catalyst under four conditions: Ar atmosphere (black), CO_2 atmosphere (red), Ar atmosphere with 2% v/v H_2O (blue), and CO_2 atmosphere with 2% v/v H_2O (green) at a scan rate of 100 mV s^{-1}. All potentials are referenced to NHE. b) Cyclic voltammograms of the precursor complex $AgBF_4$ under Ar and CO_2 atmospheres.

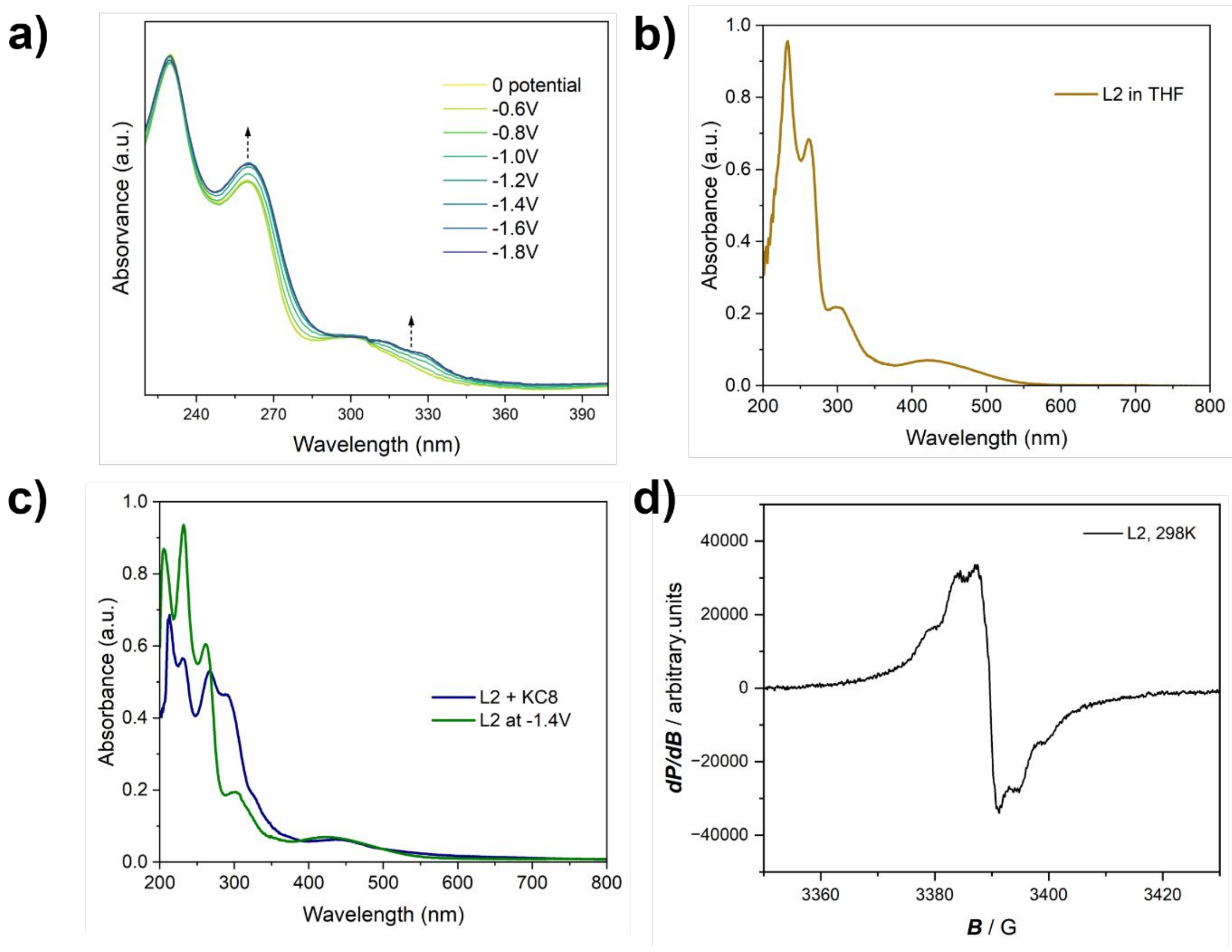

Figure S23. **a)** In situ UV-vis spectroelectrochemistry of **L2** in THF at applied potentials from 0 V to −1.8 V, showing progressive increase in absorbance at ~265 nm and emergence of a new feature at ~325 nm with increasing cathodic potential, indicative of $BIAN^{\bullet-}$ radical anion formation. **b)**UV-vis absorption spectrum of **L2** in THF, showing characteristic BIAN π–π* transitions below 350 nm **c)** UV-vis absorption spectra comparing **L2** reduced chemically with KC_8 (blue) and electrochemically at −1.4 V vs NHE (green) in THF, demonstrating close agreement between the two reduction methods and confirming chemically reversible ligand-centred reduction. **d)** X-band EPR spectrum (first derivative, dP/dB) of the KC_8-reduced **L2** ligand radical anion (**[$L2^{\bullet-}$]**, 298 K, THF).

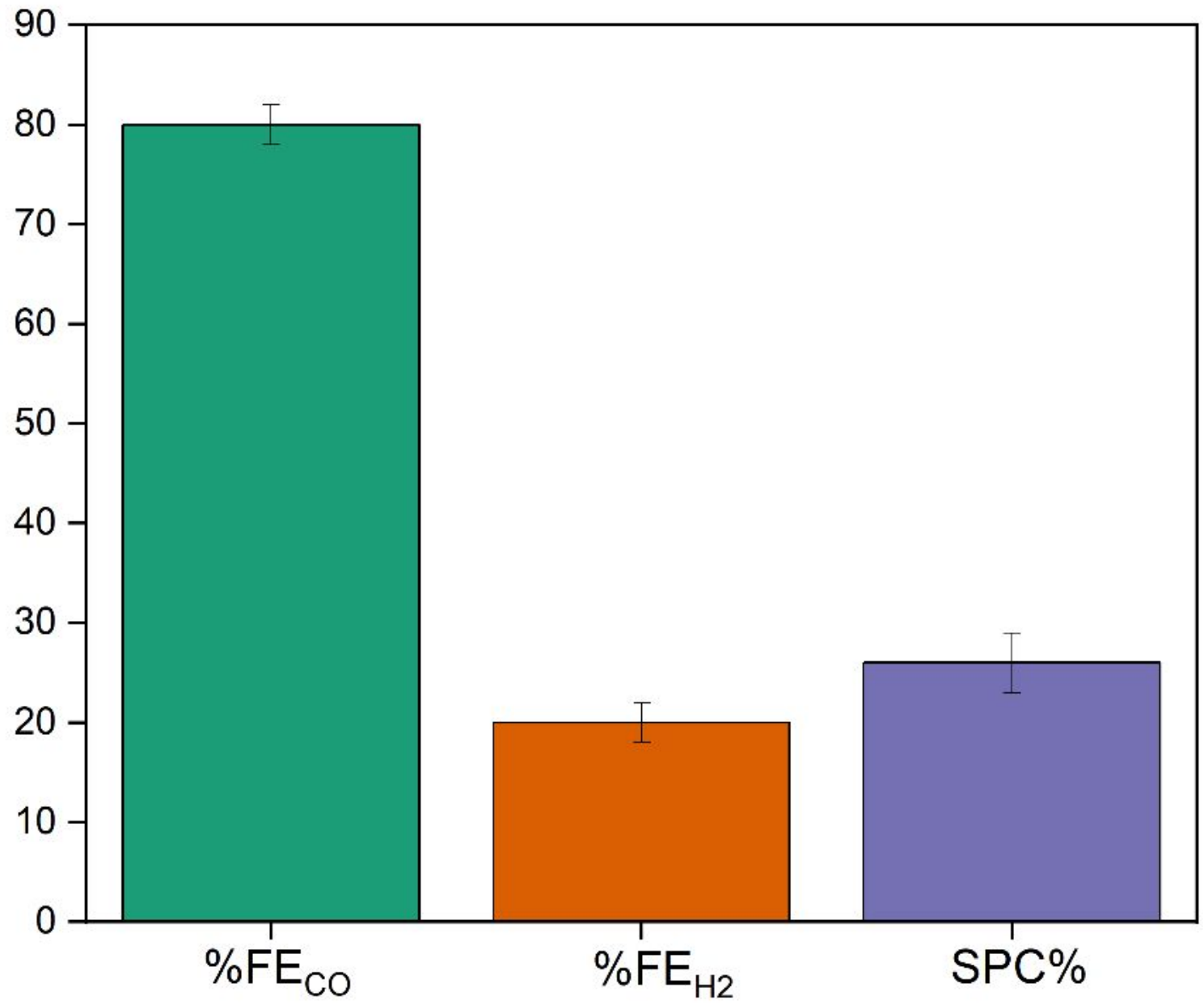

Figure S24. Faradaic efficiencies for CO and H_2 as well as single-pass-conversions of catalyst **Ag2** in an optimized zero-gap electrochemical cell environment (area = 9 cm^2) at 300 mA/cm^2.
